# Element selection for crystalline inorganic solid discovery guided by unsupervised machine learning of experimentally explored chemistry

**DOI:** 10.1038/s41467-021-25343-7

**Published:** 2021-09-21

**Authors:** Andrij Vasylenko, Jacinthe Gamon, Benjamin B. Duff, Vladimir V. Gusev, Luke M. Daniels, Marco Zanella, J. Felix Shin, Paul M. Sharp, Alexandra Morscher, Ruiyong Chen, Alex R. Neale, Laurence J. Hardwick, John B. Claridge, Frédéric Blanc, Michael W. Gaultois, Matthew S. Dyer, Matthew J. Rosseinsky

**Affiliations:** 1 Department of Chemistry, University of Liverpool, Liverpool, UK; 2 Stephenson Institute for Renewable Energy, University of Liverpool, Liverpool, UK; 3 Leverhulme Research Centre for Functional Materials Design, Materials Innovation Factory, University of Liverpool, Liverpool, UK

**Keywords:** Electronic materials, Energy modelling

## Abstract

The selection of the elements to combine delimits the possible outcomes of synthetic chemistry because it determines the range of compositions and structures, and thus properties, that can arise. For example, in the solid state, the elemental components of a phase field will determine the likelihood of finding a new crystalline material. Researchers make these choices based on their understanding of chemical structure and bonding. Extensive data are available on those element combinations that produce synthetically isolable materials, but it is difficult to assimilate the scale of this information to guide selection from the diversity of potential new chemistries. Here, we show that unsupervised machine learning captures the complex patterns of similarity between element combinations that afford reported crystalline inorganic materials. This model guides prioritisation of quaternary phase fields containing two anions for synthetic exploration to identify lithium solid electrolytes in a collaborative workflow that leads to the discovery of Li_3.3_SnS_3.3_Cl_0.7._ The interstitial site occupancy combination in this defect stuffed wurtzite enables a low-barrier ion transport pathway in hexagonal close-packing.

## Abstract (web summary)

Machine learning has the potential to significantly speed-up the discovery of new materials in synthetic materials chemistry. Here the authors combine unsupervised machine learning and crystal structure prediction to predict a novel quaternary lithium solid electrolyte that is then synthesized.

## Introduction

Researchers select new chemistries to investigate based on hypotheses generated from their understanding, for example, in choosing specific combinations of elements (referred to as phase fields hereafter) to explore to synthesise new materials. Here it is the choice of the chemical elements defining the phase field that is decisive in determining the outcomes of the study, as this delimits the attainable compositions and structures: clearly, subsequent decisions are critically important but the bounds are set by the choice of a phase field through selection of a subset of elements from those available in the periodic table. In solid-state materials chemistry, information on stable crystalline compounds is available at scale (>200,000 entries in the Inorganic Crystal Structure Database, ICSD)^1^. The factors underlying the stability, whether kinetic or thermodynamic, of these compounds are many and complex, reflecting the diverse forms of bonding interaction between their constituent elements. It is, however; difficult for researchers to hold thousands of such prior examples in mind whilst deciding which of the large numbers of unexplored phase fields to study^2^. Synthetic exploration of phase fields yields new structures and materials compositions that drive condensed matter science^3–6^. We aggregate the information in ICSD on reported phases to define those phase fields that contain synthetically isolated compounds, rather than individual materials compositions, and thus guide element selection for synthesis.

There has been a surge of machine learning (ML) studies that aim to extract the underlying patterns of chemistry available from ICSD^7–10^, based on the knowledge of the composition and structure at the level of an individual material. These statistical methods represent materials as multidimensional vectors (feature vectors). Significant progress has been achieved in deriving the required features^1,11^. Both the supervised and unsupervised learning strategies have been successfully applied to a wide variety of structure-property relationships in chemical sciences. Supervised learning^8,12–14^ infers relationships between materials’ features and materials’ properties, and requires large hand-labelled datasets for training. Unsupervised ML, on the other hand, infers underlying patterns of chemical knowledge in the absence of human-labelled data^15,16^. We report a collaborative ML-human expert workflow, in which unsupervised learning addresses the combinatorial problem of the discovery of new materials in the high-level formulation required by synthetic chemists to recognize the patterns at the level of element combinations that define those phase fields known to contain synthetically isolated crystalline compounds. This goes beyond the traditional focus at the level of individual materials to support the decisions made in identifying new chemistries to explore. We present a neural network model that tackles this distinct prediction task to allow prioritisation of new phase fields for investigation according to the extent to which their particular element combinations reflect those chemistries that lead to phase stability in reported materials. We use this model to rank unexplored quaternary two anion phase fields for experimental investigation as lithium-ion conductor candidates, noting the relative under-representation of multiple anion compounds as a class of crystalline materials^17,18^ and their importance, notwithstanding their overall rarity, as solid electrolytes that arises from a range of properties^19,20^. Machine learning-assisted researcher assessment of the candidate element combinations then identified the Li-Sn-S-Cl field. Probe structure computation^21,22^ targeted a region within this field for synthetic exploration that afforded the defect stuffed wurtzite Li_3.3_SnS_3.3_Cl_0.7_. Structural and dynamical analysis of this phase demonstrated a new pathway for lithium transport in hexagonal close-packing (hcp).

## Results and discussion

**Fig. 1:**
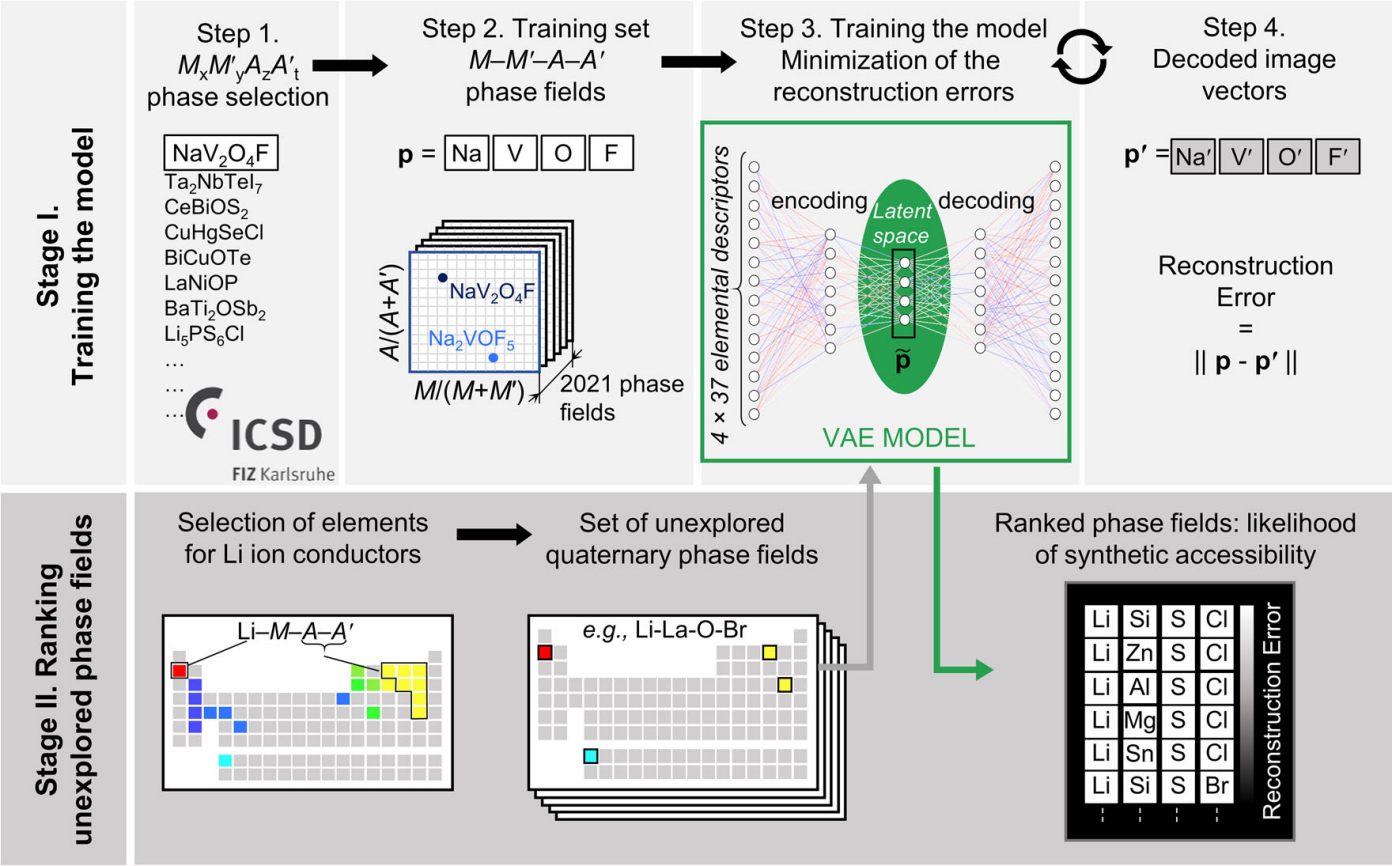
Schematics of the method for phase-field ranking for synthetic exploration. Stage I: ML model training from ICSD data of reported chemical systems in four steps: **1** quaternary phases with two anions are collected, including, for example, NaV_2_O_4_F.; **2** The individual phases (e.g., both the NaV_2_O_4_F and Na_2_VOF_5_) are aggregated to define those phase fields that contain reported two anions quaternaries. Each phase field is represented as a 148-dimensional (four elements × 37 elemental features) vector **p**; **3** the VAE^29^ is used to reduce (encode) **p** into a four-dimensional latent vector, $\tilde{{{{{{\bf{p}}}}}}}$; **4** the VAE decodes $\tilde{{{{{{\bf{p}}}}}}}$ back to a 148-dimensional image vector, **p**′. During training the VAE tunes the weights and biases of its neural networks to minimize the reconstruction errors—the Euclidean distances between the original, **p**, and decoded, **p**′, vectors. When exploring a new quaternary phase field with two anions (Stage II), e.g., Li-La-O-Br, we use the trained VAE model to encode the new phase field into the latent space and then decode it while measuring its reconstruction error. The reconstruction error of a candidate phase field captures the degree of its deviation from reported chemical systems, and enables the ranking of unexplored phase fields for synthetic exploration.

Machine learning (ML) models are powerful tools to study multivariate correlations that exist within large datasets but are hard for humans to identify^16,23^. Our aim is to build a model that captures the chemical interactions between the element combinations that afford reported crystalline inorganic materials, noting that the aim of such models is efficacy rather than interpretability, and that as such they can be complementary guides to human experts. The model should assist expert prioritization between the promising element combinations by ranking them quantitatively. Researchers have practically understood how to identify new chemistries based on element combinations for phase-field exploration, but not at significant scale. However, the prioritization of these attractive knowledge-based choices for experimental and computational investigation is critical as it determines substantial resource commitment. The collaborative ML workflow^24,25^ developed here includes a ML tool trained across all available data at a scale beyond that, which humans can assimilate simultaneously to provide numerical ranking of the likelihood of identifying new phases in the selected chemistries. We illustrate the predictive power of ML in this workflow in the discovery of a new solid-state Li-ion conductor from unexplored quaternary phase fields with two anions. To train a model to assist prioritization of these candidate phase fields, we extracted 2021 *M*_*x*_*M*′_*y*_*A*_z_*A*′_*t*_ phases reported in ICSD (Fig. 1, Step 1), and associated each phase with the phase fields *M*-*M*′-*A*-*A*′ where *M*, *M*′ span all cations, *A*, *A*′ are anions {N^3−^, P^3−^, As^3−^, O^2−^, S^2−^, Se^2−^, Te^2−^, F^−^, Cl^−^, Br^−^, and I^−^} and *x, y, z, t* denote concentrations (Fig. 1, Step 2). Data were augmented by 24-fold elemental permutations to enhance learning and prevent overfitting (Supplementary Fig. 2).

ML models rely on using appropriate features (often called descriptors)^26^ to describe the data presented, so feature selection is critical to the quality of the model. The challenge of selecting the best set of features among the multitude available for the chemical elements (e.g., atomic weight, valence, ionic radius, etc.)^26^ lies in balancing competing considerations: a small number of features usually makes learning more robust, while limiting the predictive power of resulting models, large numbers of features tend to make models more descriptive and discriminating while increasing the risk of overfitting. We evaluated 40 individual features^26,27^ (Supplementary Fig. 4, 5) that have reported values for all elements and identify a set of 37 elemental features that best balance these considerations. We thus describe each phase field of four elements as a vector in a 148-dimensional feature space (37 features × 4 elements = 148 dimensions).

To infer relationships between entries in such a high-dimensional feature space in which the training data are necessarily sparsely distributed^28^, we employ the variational autoencoder (VAE), an unsupervised neural network-based dimensionality reduction method (Fig. 1, Step 3), which quantifies nonlinear similarities in high-dimensional unlabelled data^29^ and, in addition to the conventional autoencoder, pays close attention to the distribution of the data features in multidimensional space. A VAE is a two-part neural network, where one part is used to compress (encode) the input vectors into a lower-dimensional (latent) space, and the other to decode vectors in latent space back into the original high-dimensional space. Here we choose to encode the 148-dimensional input feature space into a four-dimensional latent feature space (Supplementary Methods). The VAE model is trained to minimize the Euclidean distances (reconstruction errors) between the original and decoded vectors in the 148-dimensional feature space (Fig. 1, Step 4). In addition, VAEs avoid overfitting by putting additional constraints arising from changes in feature distribution in the latent space to make it better organized, e.g., by favoring similar decoding for nearby vectors in the latent space. In this way, VAEs ensure that the lower-dimensional vectors in latent space capture the necessary information to properly describe and discriminate between entries in the training set. Because the training set in our case includes only phase fields where quaternary compounds are experimentally reported in the ICSD, we expect our model to learn the encoding biased to those phase fields that contain synthetically accessible quaternary compounds. The reconstruction error then captures the degree to which data deviate from the learned model, and therefore can be used to rank new input data entries by their similarity to general trends in the training data^30,31^; in our case, between the candidate phase fields and those phase fields that contain compounds reported in ICSD. Similarly to one-class classifiers, the VAE is trained on positives, hence it ranks based on the likelihood of positive outcomes, rather than the absence of negative outcomes. This is appropriate for the materials discovery problem, as we can only make positive statements with certainty about observed compounds, while the absence of reported compounds in a particular phase field might simply reflect the synthetic pathways explored to date: there are only strong positive examples available. Validation of the VAE thus combines statistical evaluation with a qualitative assessment of the tool based on its role in supporting the successful exploration of new chemistries.

In the second stage (Stage II, Fig. 1), the VAE model built in Stage I is used to quantify the likelihood that selected unexplored quaternary two anion phase fields contain stable compounds. Focusing the search on new Li-ion conductors, the choice of anions and cations can be narrowed down by their physical characteristics. For example, we avoid transition metals, which may introduce undesired redox properties, as well as toxic or particularly scarce elements. Hence, we select the candidate phase fields Li-*M*-*A*-*A*′, where *M* = {B, Mg, Al, Si, P, K, Ca, Zn, Sr, Y, Zr, Sn, Ba, Ta, La} represent cations, *A* and *A*′ represent anions, {N, O, S, F, Cl, Br, I}, forming 303 unexplored phase fields in total. This constitutes a set of candidate chemistries that are attractive to researchers because they respect the criteria used to select them but which require prioritization for investigation. The VAE is trained to rank these candidates by their reconstruction error (RE - the key metric for autoencoders across a variety of applications^31–34^) and thus provides quantitative guidance for consideration by the researcher in the choice between chemistries that are qualitatively of interest. A full list of the ranked candidate phase fields is given in Supplementary Table 3. This list suggests the most promising candidates with a high likelihood of forming isolated compounds, whose detailed theoretical and experimental future study should be prioritized.

**Fig. 2:**
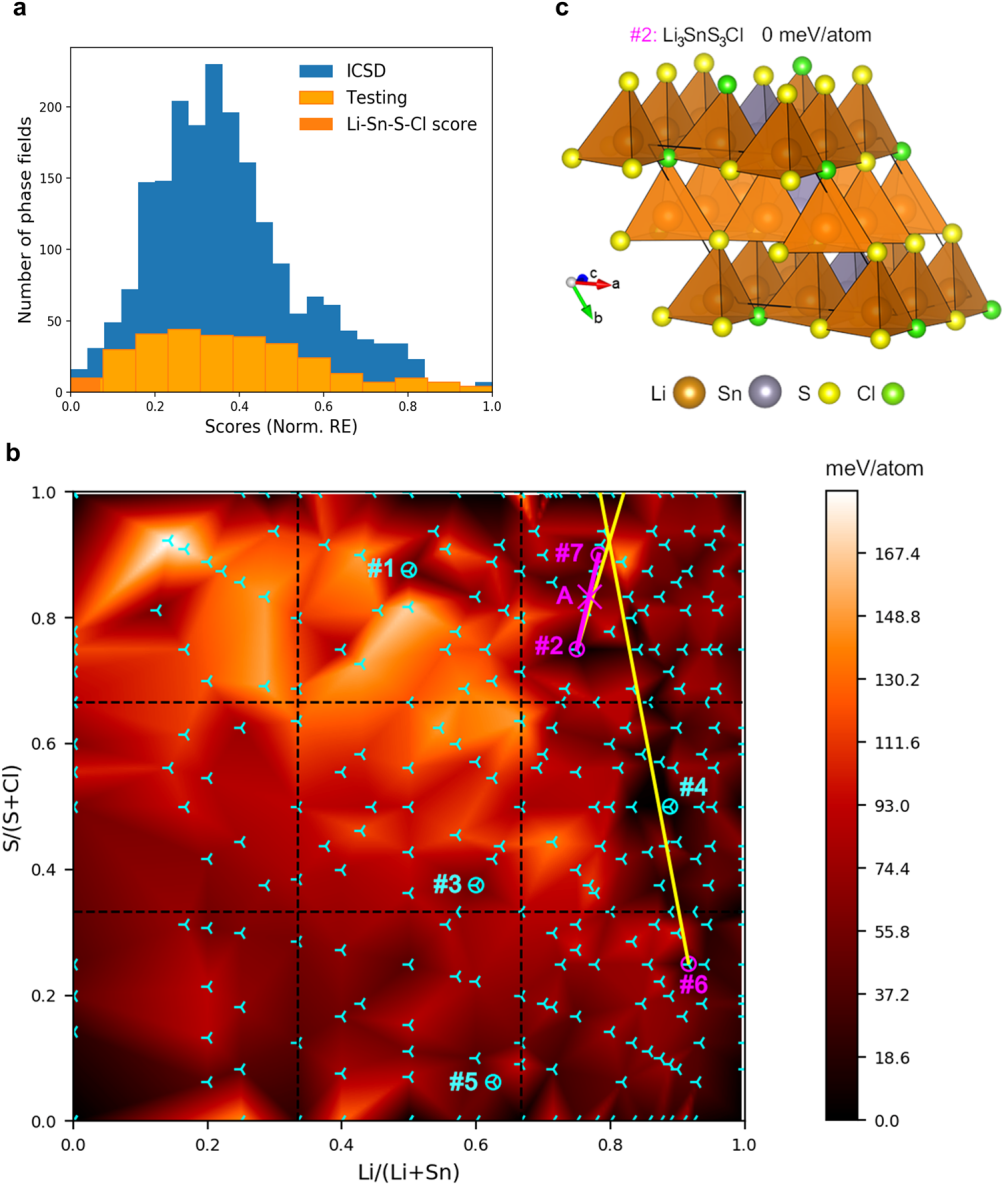
Outcome of the phase-field ranking method: from selection of elements to composition. **a** Distributions of the normalized reconstruction errors (scores) for training (ICSD-based, blue) and test (unexplored phase fields, orange) datasets, with the range of scores segmented according to^69^ Supplementary Equation 1. The Li-Sn-S-Cl phase field was chosen from the best-reconstructed candidates (dark orange). **b** Computational and experimental exploration of Li-Sn-S-Cl. Computed energies for 244 probe structures (cyan tripods) and interpolated formation energy above the convex hull^8^ (color bar) guide the choice of compositions for experimental synthesis (circled tripods) (Supplementary Table 5). Samples #1 to #6: first synthesis results. Cyan circles: formation of known compounds. Magenta circles: formation of Phase A (new phase) and known compounds (#2, #6). Phase A was identified by synthesizing sample #7 deduced from relative amounts of impurities in samples #2 and #6 (Supplementary Methods). Phase A (magenta cross) was then purified by targeting one composition on the Li_3.6_SnS_3.6_Cl_0.4_— Li_4_SnS_4_ line (magenta line). **c** Predicted probe structure Li_3_SnS_3_Cl (#2) with 0 meV/atom above the convex hull, showing the wurtzite-type structure with coupled anion and cation order.

The distribution of normalized reconstruction errors (Fig. 2a) illustrates that the model is able to reconstruct efficiently most of the reported phase fields, as 79.5% have a normalized RE below 0.5. To validate the model, we hold out five different validation datasets (each consisting of 20% of the available data from ICSD), while using the remaining 80% of data to train five different VAE models. We then assess how the validation datasets are reconstructed using the corresponding VAEs. Using this method, on average, 79.8% of the phase fields in the validation datasets have normalized reconstruction errors below 0.5. In addition to conventional validation of unsupervised methods, we also ascertain that the distributions of RE persist across the validation sets, ICSD training data, and the testing data of unexplored phase fields (Supplementary Fig. 3).

The resulting ranking of unexplored elemental combinations allows us to narrow down the search to the most promising phase fields, justifying subsequent application of computationally- and experimentally expensive research techniques. To evaluate the model, we focus on the cluster of data with the smallest reconstruction errors in the test dataset (highlighted with dark orange in Fig. 2a). Among these promising phase fields, a new composition was discovered recently (not included in our training data) in the Li-P-S-O phase field^35^ (ranked #8). Three further examples of the realization of highly ranked chemistries are recently available^36,37^ (Supplementary Fig. 18-20). Among the top five unexplored phase fields, the highest Li-ion conductivity for ternary phases underlying the candidate quaternaries was reported for Li_0.8_Sn_0.8_S_2_^38^ (Supplementary Table 4). This numerical ranking guided our choice of the Li-Sn-S-Cl field (ranked #5, Supplementary Table 3) for experimental investigation.

The VAE quantifies understanding of which element combinations are likely to lead to new isolated compounds, and reflects that understanding well enough to build researcher trust in the resulting ranking through the viability of the highly scored element combinations. This trust is essential to allow confident use of the tool in choosing within the attractive candidate set of 303 phase fields. The numerical ranking of chemistry by the VAE can be used with other numerical criteria (in this case the Li-ion conductivity of ternary phases underlying the candidate quaternaries) to support researcher decisions. The selection of the Li-Sn-S-Cl field from multiple attractive, viable candidate chemistries is a typical decision required in synthetic materials chemistry.

We identified the optimal regions within the selected quaternary for synthetic exploration with high-throughput probe structure prediction^21,22^. This computes energies for structural models with cell sizes up to 13 × 13 × 13 Å^3^ aiming to capture the accessible bonding motifs at the chosen composition, thus approximating the lowest accessible energy at that composition and highlighting regions of chemical space where previously unreported phases are likely to be found. We employ Crystal Structure Prediction (CSP) for 244 different compositions in the Li-Sn-S-Cl phase field (cyan tripods in Fig. 2b) with the complementary algorithms of basin-hopping ChemDASH^22,39^, which explores the energy landscape, with starting ionic configurations based on hcp and rhombohedral lattices and evolutionary XtalOpt^40^ exploring random and mutated (hybrid) ionic configurations drawn from a population of structures, spanning in total up to 1200 structures for each composition to maximize coverage of possible ionic arrangements. Both methods are coupled with VASP^41^ for energy computation at the DFT level of accuracy (cf. Methods). We use these energies and those of reported compounds to calculate the convex hull^8^, which defines thermodynamic stability at 0 K (Fig. 2b). Regions close (≤50 meV per atom) to the convex hull suggest a range of potentially stable compositions for experimental synthesis. This includes a composition with an energy on the convex hull, Li_3_SnS_3_Cl (Fig. 2b, Composition #2), i.e., predicted independently by both CSP methods to be thermodynamically stable at 0 K. Its composition, and its predicted wurtzite-related structure, with one of the two tetrahedral sites selectively occupied and anion order around the distinct cations (Fig. 2c), are closely related to the experimentally discovered phase that is discussed herein.

We sample the experimental points from across the phase field, while favoring compositions with a predicted low formation energy, aligned with the motivation of the workflow to focus and thus minimize synthetic work needed to identify new phases. Maximizing the coverage with a minimum number of samples, we choose the lowest energy point for synthesis from each sector of the phase field on a 3 × 3 grid (Fig. 2b), discarding compositions with low lithium content (<10 mol%). This leads to the selection of six samples (#1-6, circled tripods in Figure 2b), which we synthesized via a high-temperature solid-state route in evacuated quartz ampoules and identified the resulting phases with X-ray diffraction (Supplementary Table 5).

**Fig. 3:**
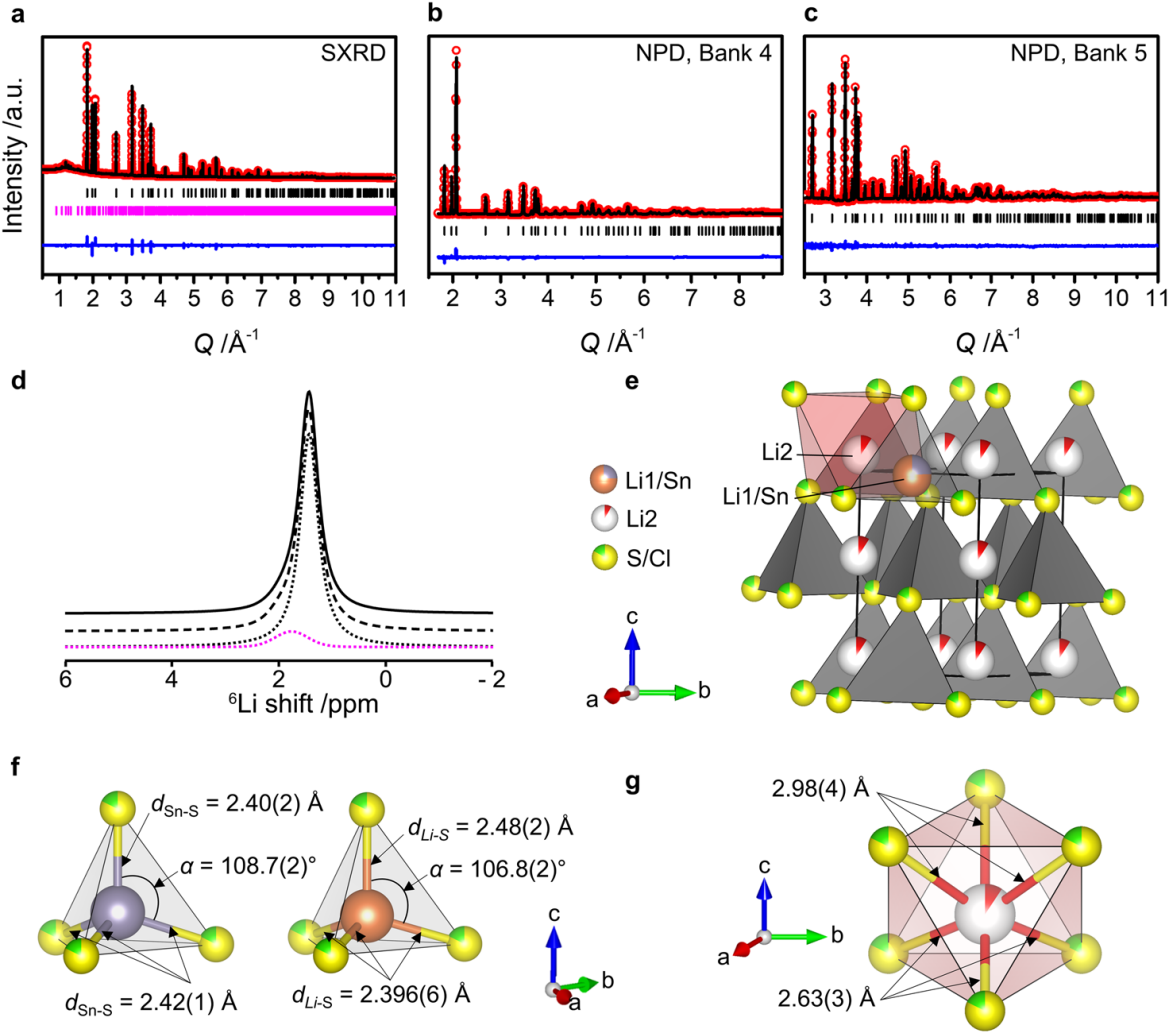
Structure determination of Li_3.3_SnS_3.3_Cl_0.7_. Final Rietveld fit against **a** SXRD data, NPD data from **b** Bank 4 and **c** Bank 5 of Polaris diffractometer with *I*_obs_ (red dots), *I*_calc_ (black line), *I*_obs_—*I*_calc_ (blue line), and Bragg reflections (black tick marks for Li_3.3_SnS_3.3_Cl_0.7_, pink tick marks for Li_4_SnS_4_ (∼4 wt% (10 mol%)). This impurity could not be detected in the NPD patterns. **d** ^6^Li MAS NMR spectrum. The experimental spectrum (full line), total fit (dashed line), spectral deconvolution (dotted lines) and orthorhombic Li_4_SnS_4_ impurity^41^ (pink dotted lines) are shown. **e** The defect stuffed wurtzite average structure of Li_3.3_SnS_3.3_Cl_0.7_ with Li1 (orange) and Sn (purple) occupying the tetrahedral site (grey) and Li2 the octahedral site (red) along with the polyhedral environments for **f** Sn and Li1 and **g** Li2. The hcp anion sublattice is shown by the mixed S (yellow) and Cl (green) sites. The combined refinement of X-ray and neutron diffraction data, where S and Cl have different scattering lengths^70^, enabled precise anion site occupancy refinement without the assumption of full site occupancy. Bulk elemental analysis of Li_3.3_SnS_3.3_Cl_0.7_ was performed using WDX and ICP-AES analysis (Supplementary Discussion) and confirmed the assumption required for the multiple source refinement of anion occupancies that S and Cl are the only elements occupying the anion sites.

For two out of the six samples (magenta circles on Fig. 2b), there is a set of Bragg reflections that does not correspond to any reported phase (Supplementary Fig. 6a, b), which we associate with a new phase denoted Phase A. From the Rietveld refinements of these two samples, we derive the relative weight fractions that are used to determine a subsequent composition close to that of Phase A (Supplementary Methods). Graphically, this corresponds to the intersection of the two yellow lines in Fig. 2b—a composition close to Li_3.6_SnS_3.6_Cl_0.4_ (Sample #7). After the reaction, this sample revealed the presence of Phase A with a single impurity of orthorhombic Li_4_SnS_4_ (Supplementary Fig. 6c) indicating that Phase A lies on the line: Li_4-*x*_SnS_4-*x*_Cl_*x*_, 0.4 < *x* < 1 (magenta line, Fig. 2b). Samples were made along the line using the same synthetic procedure (Supplementary Methods). For x = 0.7, no impurities were detected by laboratory XRD, therefore, the composition of Phase A was identified as Li_3.3_SnS_3.3_Cl_0.7_ (magenta cross, Fig. 2b). It is important to note that, as neither element ratios nor cell sizes are bounded within a phase field, it is not possible to target the exact composition for synthesis. This reinforces the importance, as the first step of the workflow, of the quantitative assessment of the selection of the phase field itself as the overall determinant of whether materials are subsequently isolated, and thus the aggregation of individual phases at the level of phase fields for model construction: identification of element combinations for study at the level of the periodic table is thus the problem addressed by the VAE model. We determine the structure of Phase A through combined Rietveld refinement of powder Synchrotron X-Ray Diffraction (SXRD) and Neutron Powder Diffraction (NPD) data (Fig. 3a–c). The reflections corresponding to Phase A were indexed to a hexagonal unit cell with lattice parameters *a* = 3.9723(3) Å and *c* = 6.3524(6) Å. Systematic absences gave the extinction symbol *P* – – *c* with the refinement of both the datasets demonstrating site occupancies within the hcp anion array consistent only with the *P*6_3_*mc* space group (Supplementary Discussion).

The diffraction-derived average structure is a defect stuffed wurtzite, where Cl and S atoms are randomly distributed on the fully occupied anionic site and the Sn and Li atoms on the fully occupied tetrahedral T^+^ interstices, with the T^−^ sites empty, while the remaining 0.3 lithium atoms per formula unit are located on the octahedral site, O (Fig. 3e). Refinement of the site occupancy factors (sof) reveals full occupancy of the anionic and the tetrahedral sites with S/Cl and Sn/Li atomic ratios of 0.823(8)/0.175(8) and 0.242(3)/0.760(3), respectively, in agreement with the expected composition. Room temperature ^6^Li Magic Angle Spinning (MAS) Nuclear Magnetic Resonance (NMR) reveals the presence of one main deshielded signal centered at 1.4 ppm (Fig. 3d), in agreement with the majority of Li occupying the tetrahedral interstices (Fig. 3f - octahedral sites are located near 0 ppm)^42^, as well as a small shoulder at 1.8 ppm attributed to a small amount (∼10 mol%) of orthorhombic Li_4_SnS_4_^43^. Interestingly, the computed probe structure for the closely related composition Li_3_SnS_3_Cl, which lies on the convex hull and thus reflects potential thermodynamic stability, also shows a wurtzite-type structure, with S^2−^ and Cl^−^ anions packed in an hcp manner, Li^+^ and Sn^4+^ occupying T^+^ interstices only, and both anions and cations ordering over these sites in a 3:1 arrangement (Fig. 2c). Here Sn^4+^ is purely co-ordinated by sulfide while Li^+^ forms both LiS_2_Cl_2_ (one third) and LiS_3_Cl tetrahedra (two-thirds).

In the experimental average structure, there is no cation or anion site ordering over diffraction length scales, but there is evidence that local anion order influences the cation coordination environments, because different average positions for Li1 and Sn on the single T^+^ site are refined from the combined diffraction data. The Li1 position is slightly displaced towards the base of the tetrahedron compared to Sn, which presents a much more regular coordination environment (Fig. 3f, Supplementary Table 7, 8) that can be associated with a predominant S-only single anion first coordination sphere driven by the higher formal cation charge and consistent with the anion ordering at the distinct cation sites in the probe structure of Li_3_SnS_3_Cl (Fig. 2c). The less regular geometry around Li1 would then be associated with the presence of both S and Cl anions in its first coordination sphere, as seen in the computed probe structure, driven by the lower formal charges of Li and Cl.

The Fourier difference map (Supplementary Fig. 7) enabled us to identify the second Li site (Li2), within the O interstice, and the refinement of the sof showed a non-negligible occupancy of 0.092(8) (Fig. 3g). This model led to an excellent fit to both datasets (Fig. 3a–c) and accounted for the remaining Li atoms, with a refined phase composition of Li_3.41(4)_SnS_3.29(3)_Cl_0.70(3)_ (Supplementary Discussion), close to the measured bulk composition Li_3.305(14)_Sn_1.000(9)_S_3.317(44)_Cl_0.6269(8)_ as determined by ICP-AES and supported by WDX analysis (Supplementary Discussion, Supplementary Fig. 8). The measured bulk composition is consistent with the presence of only S and Cl as anion-forming elements, determined by ICP-AES and WDX, and with the nominal composition Li_3.3_SnS_3.3_Cl_0.7_, which we use throughout the paper on the basis of these bulk and phase analyses.

Li_3.3_SnS_3.3_Cl_0.7_ is compositionally related to the known material Li_4_SnS_4_, which has been reported to exhibit crystalline dimorphism, with both the structures consisting of hcp-type packing of the S^2−^ anions^43,44^ but it has a quite different structure. The thermodynamically stable Li_4_SnS_4_ phase crystallizes in an orthorhombic space group with a γ-Li_3_PO_4_ structure type^43^, while the low-temperature phase (prepared by mechanosynthesis or wet chemistry) is metastable and transforms irreversibly into the orthorhombic phase when heated above 350 °C^44,45^. Low-temperature Li_4_SnS_4_ has regular hcp S^2−^ packing in space group *P*6_3_*/mmc* but is not a wurtzite, as the Sn cations partially occupy both the T^+^ and the T^−^ sites in the hcp anion array i.e., it is quite unlike Li_3.3_SnS_3.3_Cl_0.7_, where only one of the two sets of tetrahedral sites are occupied, lowering the average structure symmetry to *P*6_3_*mc*. Cationic substitution of As and Sb for Sn in stable Li_4_SnS_4_ were reported previously, and shown to maintain the orthorhombic structure^44,45^. Comparing these quaternary chemistries that emerge from the Li-Sn-S ternary, Cl is a more sustainable element than As or Sb^46^, which represent cation substitution into a single anion material, and the resulting two anion chemistry leads to a well-defined original structure. The distinction between the single and multiple anion chemistry is demonstrated by the different structures accessed. The new two anion structure represents a platform for subsequent cation substitution.

In Li_3.3_SnS_3.3_Cl_0.7_, the introduction of the second anion, Cl^−^, stabilized the high symmetry hexagonal sulfide packing to permit high synthesis temperatures that afforded sufficient crystallinity (coherent scattering domain of 200(10) nm, Supplementary Methods) to locate and refine Li positions and occupancies, producing the first full structural model of a Li-ion conducting defect stuffed wurtzite.

Here, the use of the VAE and CSP tools in the collaborative workflow enabled targeting the exploration of an entire phase field that resulted in the discovery of a new phase. The isolation of such a phase of narrow compositional width with a specific and high content of the second anion would not be facilitated by low-level doping of the known Li_4_SnS_4_ structure, which is the most common approach. Although it is not possible to prove exhaustively that other cations would not also lead to the formation of this new phase, because the VAE is trained only on positive examples, the successful outcome supports the practical use of the VAE in assisting selection of element combinations for synthetic exploration, and demonstrates the consequential nature of the choice of the Li-Sn-S-Cl phase field from the other candidates in the test set that also offer attractive chemistries.

**Fig. 4:**
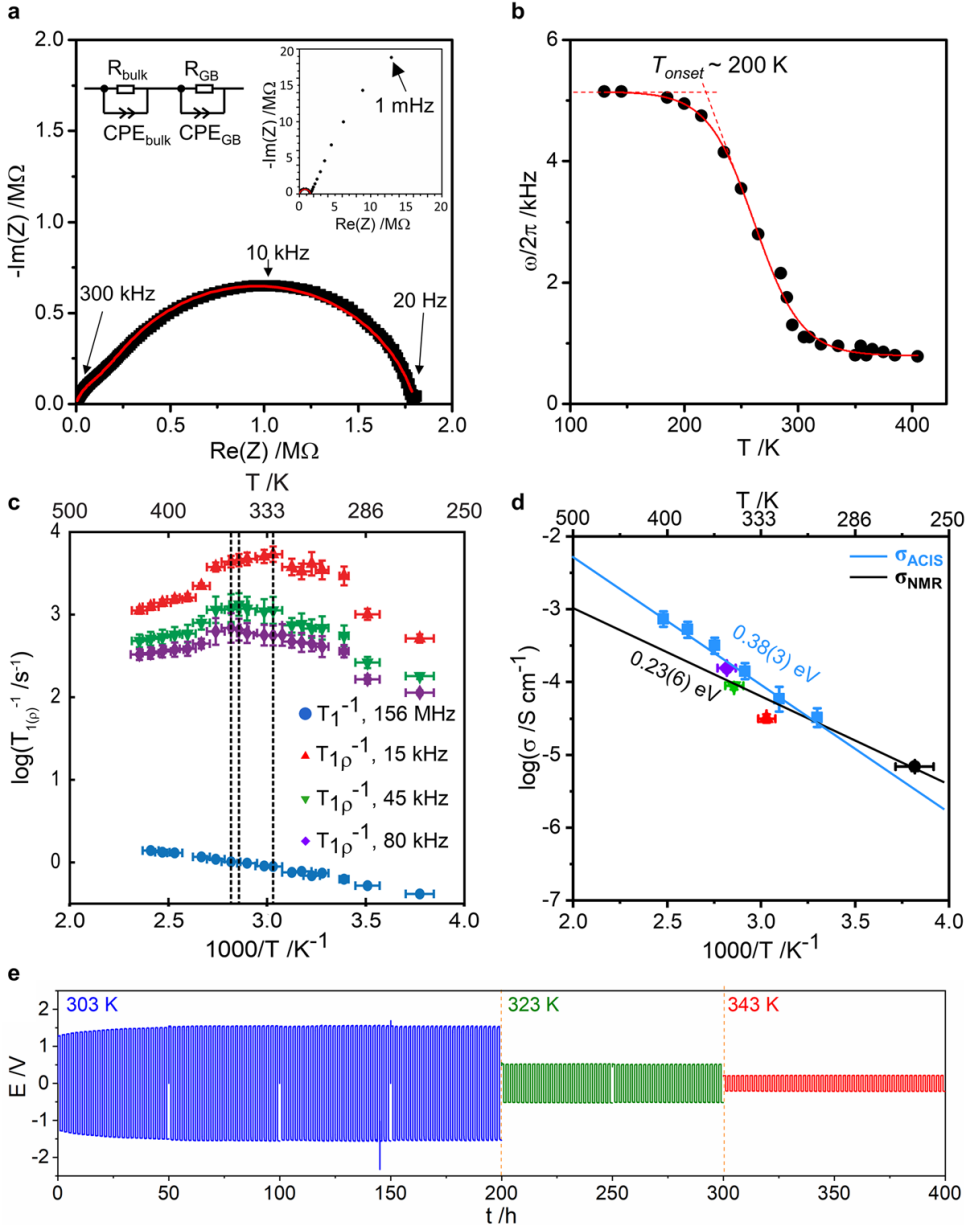
Lithium conductivity and dynamics in Li_3.3_SnS_3.3_Cl_0.7_. **a** Room temperature (303 K) Nyquist plot of impedance response and the fit (red line) using the equivalent circuit above, showing the two contributions to the conductivity, CPE constant phase element, R resistance, and GB grain boundary. Inset shows the impedance spectra over an extended frequency range. **b** Temperature dependence of static ^7^Li NMR line width as a function of temperature. The solid line is a sigmoidal regression fit and is a guide to the eye. Increase in Li motion starts at *T*_*onset*_. **c** Arrhenius plot of spin-lattice relaxation (SLR) rates in the laboratory frame (T_1_^−1^) at a Larmor frequency of 156 MHz (blue circle) and in the rotating frame (T_1ρ_^−1^) at spin-lock frequencies ω_1_/2π of 15 (red triangle), 45 (green inverted triangle) and 80 kHz (purple diamond). The colored ticks on the upper *x*-axis represent the temperatures of the T_1ρ_^−1^ maxima. The error bars associated with the temperature are calculated from the broadening of the isotropic peak of the chemical shift thermometer Pb(NO_3_)_2_ (Supplementary Methods), while the errors in T_1_^−1^ and T_1ρ_^−1^ are obtained from the output of the fittings of Supplementary Equations 2 and 3 respectively. **d** Arrhenius plot of the bulk conductivity measured by ACIS (blue circles) and calculated from NMR jump rates using Supplementary Equation 5 from line narrowing experiments (black circle, panel b) and SLR rates T_1ρ_^−1^ (panel c) using the same color coding. The corresponding linear fits using an Arrhenius law are given in blue and black lines. The errors obtained in σ_NMR_ were derived from the propagation of the various errors associated with Supplementary Equation 5. **e** Galvanostatic Li plating/stripping voltage profile of a symmetric Li | Li_3.3_SnS_3.3_Cl_0.7 _| Li cell measured at 10 µA cm^−2^ at 303, 323, and 343 K.

We measure lithium-ion conductivity on a pressed pellet by AC-impedance spectroscopy (ACIS). The Nyquist plot shows two overlapping semicircles in the high-frequency region (Fig. 4a) and a Warburg tail in the low-frequency region (inset, Fig. 4a), indicating that the material is a typical ion conductor. The bulk and grain boundary conductivities are extracted from fitting Nyquist plots with an equivalent circuit (Fig. 4a, Supplementary Discussion). The electronic conductivity is determined by DC polarization measurement (Supplementary Discussion, Supplementary Fig. 10). At room temperature, the total, bulk, and electronic conductivity are *σ*_tot _= 2.4(1) × 10^−6^ S cm^−1^, *σ*_bulk_ = 3.2(3) × 10^−5^ S cm^−1^, and *σ*_e_ = 1.3(6) × 10^−8^ S cm^−1^, respectively. The electronic contribution to the conductivity is negligible, and accounts for only around 1% of the total conductivity. The temperature dependence of *σ*_bulk_ followed an Arrhenius behavior (Fig. 4d, blue curve), with an activation energy, *E*_a_, of 0.38(3) eV. The bulk conductivity of Li_3.3_SnS_3.3_Cl_0.7_ is slightly lower than the reported total conductivities of orthorhombic Li_4_SnS_4_ (*σ*_tot_ = 7.0 × 10^−5^ S cm^−1^)^43^ and hexagonal Li_4_SnS_4_ (*σ*_tot_ = 1.1 × 10^−4^ S cm^−1^)^44^. Moreover, galvanostatic Li plating/stripping over 400 h in a Li | Li_3.3_SnS_3.3_Cl_0.7 _| Li symmetric cell and the ex situ structural characterization confirm the back-and-forth Li^+^ transport through the Li_3.3_SnS_3.3_Cl_0.7_ solid electrolyte, as well as across the Li_3.3_SnS_3.3_Cl_0.7_ | Li^0^ interface (Fig. 4e, Supplementary Discussion, Supplementary Fig. 12), and good chemical compatibility between the Li_3.3_SnS_3.3_Cl_0.7_ solid electrolyte and Li metal (Supplementary Fig. 13, 14). An initial slight increase in plating/stripping overpotentials in the first 25 cycles (50 h) suggests the evolution of a growing interphase layer (Supplementary Discussion, Supplementary Fig. 12). Thereafter, no further overpotential increase is observed (even at higher temperatures), indicating a suppression of further interfacial reactions. Ex situ XRD patterns (Supplementary Fig. 13) and Raman spectra (Supplementary Fig. 14) of the cycled bulk pellet show the starting structure of Li_3.3_SnS_3.3_Cl_0.7_ is well maintained after 400 h cycling. This result is in contrast with the chemical incompatibility between Sb- and As-doped Li_4_SnS_4_ single anion materials and Li metal^46,47^. The new phase highlights an effective route to enhance the interfacial stability of a solid electrolyte against Li by tailoring the chemical composition and structure with the use of two anions^48^. In general, the bulk stability of the quaternary ionic conductor as studied here against lithium metal depends on the formation energy of its corresponding decomposition products^49^. An unfavorable decomposition energy is thus expected for the current Li_3.3_SnS_3.3_Cl_0.7_, which is enabled by the introduction of the second anion. The interfacial stability against lithium may arise from the formation of some initial decomposition species (Supplementary Fig. 14) that are thermodynamically stable with lithium metal (such as LiCl) and electronically insulating, which kinetically inhibit a further decomposition of the solid electrolyte.

We record the ^7^Li NMR spectra and spin-lattice relaxation rates (SLR) of Li_3.3_SnS_3.3_Cl_0.7_ to provide further insight into the dynamics on the MHz and kHz timescale (Methods, Supplementary Discussion). The temperature dependence of the full width at half maximum of the ^7^Li static NMR spectra (Fig. 4b) reveals line narrowing starting at around 200 K upon heating, and hence an increase in Li motion at this temperature. The inflection point of this temperature-dependent line width defines the Li^+^ ion jump rate, τ^−1^, which is of the order of the line width in the rigid lattice regime (∼5 kHz), yielding a value of ∼3.3(2) ×  10^4^ s^−1^ at *T* ~250 K. The SLR rates are purely induced by diffusion processes here and their increase and decrease in the rotating frame (*T*_1ρ_^−1^) with increasing temperature are characteristic of the slow and fast motion regime, respectively^49^. Maxima in Fig. 4c are observed when Li^+^ jump rates, τ^−1^, are on the order of the probe spin-lock frequency ω_1_ and obey the relationship 2ω_1_ ≈ τ_c_^−1^ (where τ_c_^−1^ is the correlation rate of the Li motion and is essentially the average τ^−1^)^50^ giving jump rate values of the order of 1.8 × 10^5^ − 1 × 10^6^ s^−1^ in the 330–355 K temperature range.

We then estimate the NMR conductivity *σ*_NMR_ from combined Nernst-Einstein and Einstein-Smoluchowski equations (Supplementary Equation 5) and extract an NMR activation barrier of 0.23(5) eV for Li-ion diffusion (Fig. 4d). This value is lower than that determined by ACIS as NMR spectroscopy determines the barrier of diffusion of Li to its neighboring site, whereas ACIS probes longer-range translational diffusion. The extrapolated conductivity value at 303 K is 2.6(7) × 10^−5^ S cm^−1^, in good agreement with the bulk conductivity value of 3.2(3) × 10^−5^ S cm^−1^ obtained from ACIS.

**Fig. 5:**
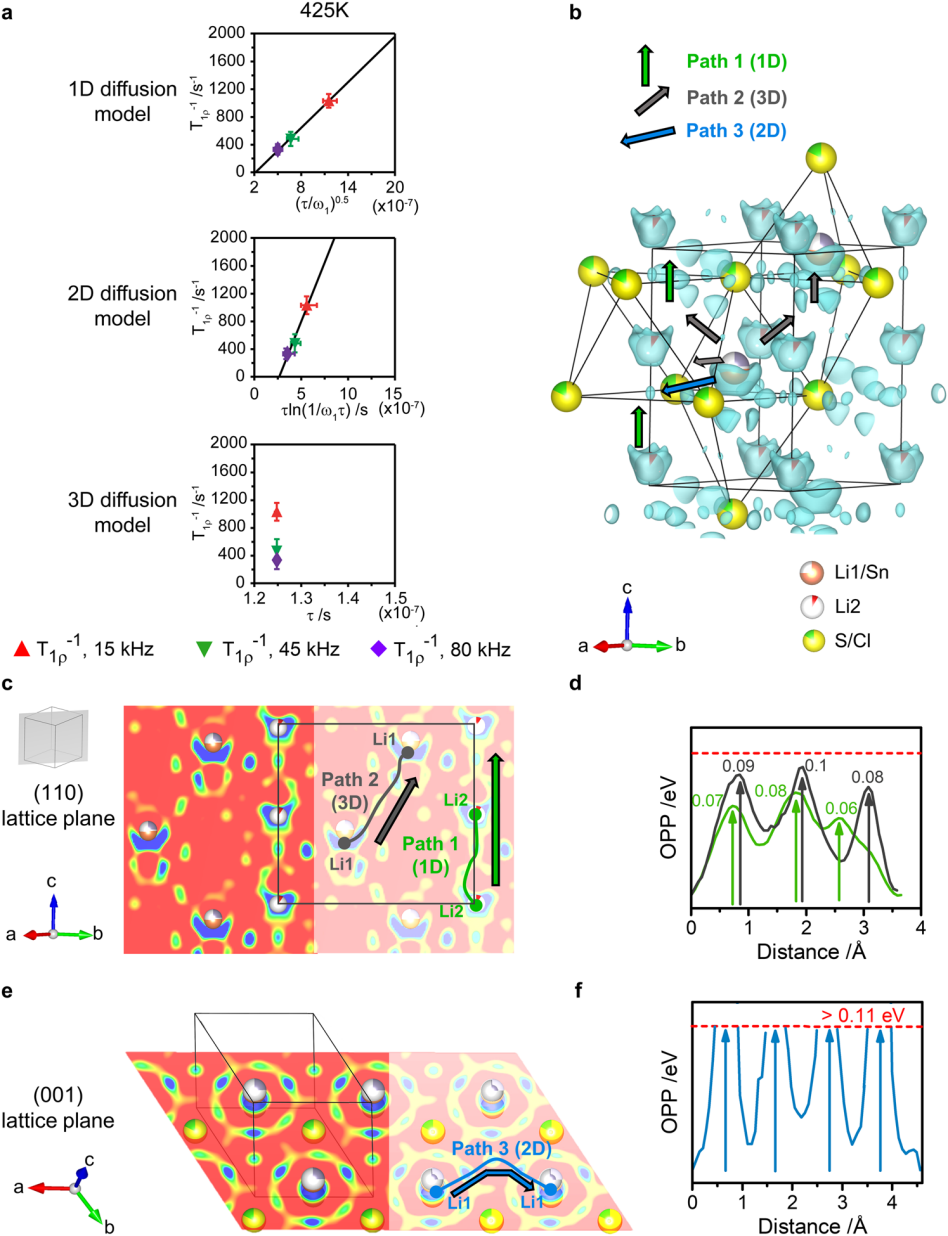
Li diffusion pathways in Li_3.3_SnS_3.3_Cl_0.7_. **a** Frequency dependence of the NMR SLR T_1ρ_^−1^ rates at 425 K for one-dimensional, two-dimensional and three-dimensional diffusion models, as a function of spin-lock frequencies ω_1_/2π of 15 (red triangle), 45 (green inverted triangle), 80 kHz (purple diamond) and average Li^+^ jump times τ from T_1ρ_^−1^ maxima (Fig. 4c). The black lines correspond to linear fits with regression factor *r*^2^ of 0.99 for 1D and 0.98 for 2D diffusion. Data for other temperatures are given in Supplementary Fig. 17. Errors in the spin-lock frequencies ω_1_ are estimated to be 10% while the errors in the correlation time τ are extracted from the fit in Supplementary Fig. 16. Errors in T_1ρ_^−1^ are obtained from the outputs of the fits to Supplementary Equation 3. **b** Nuclear density reconstructed by the maximum entropy method and highlighted potential diffusion pathways (Path 1 (1D): green arrow, Path 2 (3D): gray arrows and Path 3 (2D): blue arrows). **c** Nuclear density in the (110) lattice plane and **d** estimated one particle potential (OPP) along the two diffusion pathways in this plane (Path 1: green line; Path 2: gray line), energy barriers are indicated on the plot in eV. **e** Nuclear density in the (001) lattice plane and **f** OPP estimated along Path 3 in this plane. The dashed red line on **d** and **f** corresponds to the maximum OPP that can be calculated given the background level of the nuclear density. Density levels on **b**, **c**, **e** are plotted in the [-0.02 fm Å^−3^; 0 fm Å^−3^] range to identify scattering from Li.

The frequency dependence of the NMR SLR rates (Fig. 4c) also provides insight into the dimensionality of the Li^+^ diffusion processes. The *T*_1ρ_^−1^ values obtained are dependent on the probe frequency ω_1_ and hence eliminate the possibility of three-dimensional (3D) diffusion^51,52^. Furthermore, plots of *T*_1ρ_^−1^ against (τ/ω_1_)^0.5^ and τln(1/ω_1_τ) that are characteristic (Supplementary Discussion) of one- (1D) and two-dimensional (2D) diffusion processes, respectively^51,52^, are shown in Fig. 5a for data obtained at 425 K (Supplementary Fig. 17 shows data at other temperatures) and indicate 1D or 2D Li^+^ diffusion.

Analysis of the periodic distribution of the scattering density provides further important experimental evidence for possible diffusion pathways and their dimensionality^50,53^. Figure 5b shows the nuclear density obtained by the maximum entropy method (MEM). Three potential diffusion pathways are highlighted: i) Path 1 (O–O): 1D diffusion from one Li2 octahedron to another through their common face along the c axis (green arrows, Fig. 5b, c), ii) Path 2 (T^+^ –T^−^–T^+^): 3D diffusion from the Li1 (T^+^) tetrahedra to one of the vacant T^−^ tetrahedra of the same layer via their common edge (3 possibilities spanning the whole (*ab*) plane, grey arrows on Fig. 5b), followed by diffusion to the T^+^ site in the layer above through their common face (along *c*, grey arrows Fig. 5b, c) and iii) Path 3 (T^+^-O-T^+^): 2D diffusion in the sulfide slab from one Li1 tetrahedron to another by passing through the edge of the T^+^ base, via the octahedral interstice and again through the edge of the adjacent Li1 tetrahedron (blue arrows, Fig. 5b,e).

The nuclear density along each diffusion pathway is linked to an energy scale (the One Particle Potential, OPP) via the Boltzmann distribution^54,55^, ρ ∼ρ_0_ × e^(−OPP/kT)^ (Fig. 5d, f). In this approximation, each ion is treated as individual Einstein oscillator, considered non-deformable by bonding, and any additional defect formation energy is neglected. OPP values are not quantitative and may not be related to other techniques, but qualitative and relative comparison within the same phase is valid and has been demonstrated^56^. Path 3 shows much higher energy barriers compared to Path 1 and 2 indicating that 2D diffusion is not dominant. As NMR also dismisses possible 3D conductivity, Path 2 with its three-dimensional nature can also be discarded. This is further reinforced by the absence of vacancies on the Li1 site preventing direct Li1-Li1 jumps as described in Paths 2 and 3. By combining NMR and MEM data, we demonstrate 1D diffusion in the defect stuffed mixed anion wurtzite, involving Li2-Li2 jumps between the partially occupied O sites along the *c* axis described by Path 1. O–O was indeed identified as the lower energy barrier pathway in a model sulfide hcp lattice^57^. However, near the wurtzite structure, as O is a nonstable site, it is often left vacant when composition allows, and the limiting mechanism is defined by diffusion through T–O shared faces with higher activation energy, therefore hindering conductivity. By using two anions to stabilize Li cation excess with both O and T sites occupied, we show that defect stuffed wurtzites should be considered as promising Li ionic conductor candidates by locating the Li cations and resolving their conduction pathways. The cation stuffing onto the O site opens the lower energy barrier O–O diffusion pathway as the main limiting transport mechanism, in contrast to tetrahedral-only materials. This is consistent with the lower activation energy of Li_3.3_SnS_3.3_Cl_0.7_ (*E*_a_ = 0.38(3) eV) compared to non-stuffed wurtzite Li(BH_4_)_1-*x*_Br_*x*_ (*E*_a_ = 0.52–0.64 eV, *σ*_tot, 313K_ = 10^−6^ S cm^−1^)^58^. 1D diffusion channels are favorable for accessing liquid-like ionic conductivity behavior, as in Li_10_GeP_2_S_12_^59^, and further tuning of the composition to increase octahedral occupancy in stuffed wurtzites would increase carrier density. More generally, O–O pathways along the *c* axis, involving face sharing of octahedra, have not yet been experimentally reported in other hcp sulfide materials (Supplementary Table 10).

Selection of the elements to combine is a foundational choice in synthetic chemistry, which is difficult because of the diversity of possible bonding and structure types, and the scale of the available options, and consequential because it determines considerable subsequent investment of experimental and computational effort. By learning the interplay between the chemical characteristics of the elements themselves in those combinations that afford synthetically accessible crystalline inorganic solids, we can support human decisions to identify and prioritize chemistries under consideration for experimental exploration. Despite the absence of strong negative data in materials synthesis, the unsupervised approach of the VAE allowed training on positive examples only. We connect the latent space of the VAE to chemical space and thus select a two anion quaternary phase field that affords a new lithium-ion conductor, because the resulting representation captures complex patterns of similarity between the known and candidate phase fields that allows these candidates to be effectively ranked. Targeting of a region within this quaternary by probe structure prediction focuses experimental investigation and enables the discovery of a new mixed anion stuffed wurtzite phase of composition Li_3.3_SnS_3.3_Cl_0.7_ that displays a Li^+^ ion diffusion pathway previously unseen experimentally in hcp anion arrays, and inaccessible to date with monoanionic chemistry.

Here the collaborative workflow supports the choice to explore the more rarely investigated two anion chemistry. The distinctive structure, resulting understanding of cation transport in close-packed anion arrays and enhanced interfacial stability to lithium metal over the cation-substituted single anion systems then reveals the impact of this change to multiple anion materials. The specific choice of chemistry to study versus other attractive, viable two anion systems was supported quantitatively by the VAE, because it provides a ranking of candidate chemistries based on data at a scale that is complementary to knowledge of human researchers: that individual highly ranked chemistries align with human understanding is a point in favour of the VAE, which reinforces and quantifies this understanding, and additionally numerically ranks such chemistries. This support came not as a standalone tool but as part of a workflow with both deterministic and probabilistic components, where the final decisions are made by researchers. The workflow could be extended to include other approaches, for example, in focussing high-throughput experimental methods on promising chemistries. The new structure and property outcomes in the five successful highly ranked chemistries to date build trust in the collaborative ML approach and emerge from detailed serial experimentation and analysis using multiple techniques.

The outcome highlights the potential automated evaluation of chemical space that starts with element combination selections made at the level of the periodic table and finishes with the prioritization of specific compositional ranges. This may identify chemistries, that, while close in the latent space of the VAE and thus highly accessible synthetically, appear to the human expert quite distinct from reported materials chemistry, accelerating exploration and discovery.

## Methods

### Variational autoencoder

The VAE model^27^ consists of two parts—an encoder, with 4 hidden layers with 148, 74, 37, 18 nodes, respectively, and a decoder, with 4 hidden layers with 18, 37, 74, 148 nodes. We add 0.5 dropout^60^ after each hidden layer. Nodes are activated with ReLU^61^. The data were processed in batches of 24 entries, and weights and biases of the model were optimized during training with the ‘Adam’ method^62^.

### Crystal structure prediction (CSP)

In CSP with ChemDASH^39^, for each composition, the structure was initialized with anions (S^2−^, Cl^1−^) located on a 2 *×* 2 *×* 2 or 3 *×* 2 *×* 2 grid in a close-packed arrangement and cations (Li^1+^, Sn^4+^) occupying the interstitial sites. Up to 600 Li-Sn-vacancy and S-Cl atomic swaps were performed from initial structures and optimized geometrically with VASP to produce candidate structures for each composition.

In CSP with XtalOpt^40^, each composition was initialized with a random structure and up to nine evolutional generations were considered with 50 mutated structures in each. The generations were created by mutations of a structure as well as by combining two-parent structures into a new structure. Mutations are direct transformations of the crystal structures—crossover, strain, nonlinear “ripple”, exchange (atomic swaps), and their combinations.

### VASP calculations

Calculations for structure prediction were based on energy calculations after geometry optimization for reference and probe structures that were performed in VASP-5.4.4^41^ with PAW pseudopotentials^63^, a 700 eV kinetic energy cutoff for plane waves, and 5 *×* 5 *×* 5 k-points sampling. 1e–10 eV threshold for total energy convergence in self-consistent runs, and 0.001 eV/Å threshold for convergence of forces were used for all computations.

### Synthesis

A sample of Li_3.3_SnS_3.3_Cl_0.7_ was synthesized by solid-state annealing in evacuated sealed quartz tubes using stoichiometric amounts of Li_2_S (Merck, 99.98 %), SnS (Alfa Aesar, 99 + %), LiCl (Merck, 99.99 %), S (Merck, 99.98 %). Precursors were weighed in order to yield a total mass of powder of approximately 300 mg. Powders were combined and mixed thoroughly with an agate pestle and mortar for 15 min, transferred into an alumina crucible, and then sealed in a quartz tube under a pressure of 10^−4^ mbar. The ampoule containing the sample was heated to 700 °C at a ramp rate of 5 °C min^−1^, held at 700 °C for 12 hours, and then cooled to room temperature at a ramp rate of 5 °C min^−1^. The resulting powder was then manually ground in order to obtain a fine powder. Precursors and resulting powders were handled in an Ar-filled glovebox (O_2_ < 1 ppm). Neutron powder diffraction (NPD) experiments were conducted on a ^7^Li-enriched sample of ^7^Li_3.3_SnS_3.3_Cl_0.7_, using ^7^Li_2_S as precursor material, which was synthesized according to the method described by Leube et al.^64^, starting from ^7^Li_2_CO_3_ (Merck, 99 % ^7^Li).

### Diffraction

Synchrotron X-ray diffraction (SXRD) was performed at the I11 beamline at Diamond Light Source (Oxfordshire, UK), with an incident wavelength of 0.82637(1) Å using a wide-angle position sensitive detector, and samples sealed in *D* = 0.3 mm glass capillaries to prevent air exposure. Time-of-flight (ToF) neutron powder diffraction (NPD) data were collected at room temperature using the Polaris instrument at ISIS neutron source (Oxfordshire, U.K.). Samples were loaded in *D* = 8 mm thin-walled vanadium cylindrical cans and sealed using an In gasket in an Argon-filled glovebox.

### AC-impedance spectroscopy

A pellet of the Li_3.3_SnS_3.3_Cl_0.7_ powder was made by uniaxial pressing 30 mg of powder in a 5 mm diameter cylindrical steel die at a pressure of 125 MPa. A relative density of 80% was obtained by this method. Au electrodes were subsequently sputter coated onto both the faces of the pellet using a Q150R Plus—Rotary Pumped Coater. A.C. impedance measurements were performed using a custom-built sample holder, in the temperature range 25–125 °C, using an impedance analyser (Keysight Technologies E4990A) in the frequency range from 12 MHz to 20 Hz (with an amplitude of 50 mV). All procedures and measurements were carried out in an Ar-filled glovebox to avoid sample decomposition in the air. For measurements at a lower frequency, AC impedance was collected from 3 MHz to 1 mHz with a voltage amplitude of 100 mV using a Biologic VSP-300 potentiostat/galvanostat on a second pellet. This second measurement was made to visualize the blocking electrode response with only access to the total conductivity, while the first measurement was used to determine and dissociate bulk and grain boundary conductivities in the function of temperature (Supplementary Discussion). The impedance spectra were fitted with an equivalent circuit using the ZView2 programme^65^. Both the measurements give the same total conductivity within error.

### Maximum entropy method (MEM) analysis

Maximum entropy calculation was performed with the programme Dysnomia^66^ using an input file containing observed structure factors from the NPD data of Bank 4 of Polaris and generated by FullProf^67^. Visualization of nuclear densities and extraction of 2D displays was then performed in the programme Vesta^68^.

### Nuclear Magnetic Resonance (NMR)

All NMR data were recorded at 9.4 T on a Bruker AVIII HD spectrometer. ^6^Li Magic Angle Spinning (MAS) NMR experiments were obtained with a 4 mm HXY MAS probe (in double resonance mode) with the X channel tuned to ^7^Li at *ω*_0_*/*2*π* (^6^Li) = 58.9 MHz and under MAS at a rate of *ω*_*r*_*/*2*π* = 10 kHz. Spectra were obtained with a pulse length of 3 *µ*s at a radiofrequency (rf) field amplitude of *ω*_1_*/*2*π* = 83 kHz. The sample was packed into a 4 mm MAS rotor under an Ar-atmosphere. Variable temperature ^7^Li static NMR experiments were recorded on a 4 mm HXY MAS probe (in double resonance mode) at and below room temperature and on a 4 mm HX High-Temperature MAS Probe above room temperature with the X channel tuned to ^7^Li at *ω*_0_*/*2*π* (^7^Li) = 156 MHz. The ^7^Li spectra were recorded with a pulse length of 1.5 µs at an rf field amplitude of *ω*_1_*/*2*π* = 83 kHz. The sample was flame sealed in glass inserts under an Argon atmosphere of 10^*−*3^ mbar. All ^6,7^Li spectra were referenced to 10 M LiCl in D_2_O at 0 ppm.

Additional experimental methods are available in the Supplementary Methods.

## Supplementary information

Supplementary Information

## Data Availability

The raw ICSD-2017 data used in this study is available at https://www.github.com/lrcfmd/PhaseFieldRanking. The distribution of the phase fields’ rankings, computed phase field’s energy profile (convex hull) and experimental data generated in this study are available via University of Liverpool data repository at http://datacat.liverpool.ac.uk/id/eprint/1157. The X-ray crystallographic coordinates for the structure reported in this study have been deposited at the Cambridge Crystallographic Data Centre (CCDC), under deposition number 2023329. These data can be obtained free of charge from The Cambridge Crystallographic Data Centre via www.ccdc.cam.ac.uk/data_request/cif.

## Code availability

The code (implementation of VAE and Phase Field Ranking method) developed for this work is available at https://www.github.com/lrcfmd/PhaseFieldRanking. 10.5281/zenodo.5113851
