# Supplementary material for: Element selection for crystalline inorganic solid discovery guided by unsupervised machine learning of experimentally explored chemistry
Source: Nat Commun. 2021 Sep 21;12:5561. doi: 10.1038/s41467-021-25343-7 (PMC8455628; doi:10.1038/s41467-021-25343-7)
Supplement: Supplementary file 1 — Supplementary Information [file 41467_2021_25343_MOESM1_ESM.pdf]

Supplementary Information

Element selection for crystalline inorganic solid discovery guided by unsupervised machine learning of experimentally explored chemistry

Andrij Vasylenko[1], Jacinthe Gamon[1], Benjamin B. Duff[1,2], Vladimir V. Gusev[1,3], Luke M. Daniels[1], M. Zanella[1], J. Felix Shin[1], Paul M. Sharp[1,3], Alexandra Morcsher[1], Ruiyong Chen[1], Alex R. Neale[1,2], Laurence J. Hardwick[1,2], John B. Claridge[1,3], Frédéric Blanc[1,2,3], Michael W. Gaultois[1,3], Matthew S. Dyer[1,3], Matthew J. Rosseinsky[1,3]*

[1]Department of Chemistry, University of Liverpool, Crown Street, L69 7ZD Liverpool, UK.

[2]Stephenson Institute for Renewable Energy, University of Liverpool, Peach Street, L69 7ZF Liverpool, UK.

[3]Leverhulme Research Centre for Functional Materials Design, Materials Innovation Factory, University of Liverpool, UK

*corresponding author

E-mail: m.j.rosseinsky@liverpool.ac.uk

Supplementary Methods

Machine Learning methodology

We treat the problem of detecting phase fields that contain stable compounds as an instance of the inlier/outlier detection problem, which is tightly connected to anomaly and novelty detection tasks as well. The overview of deep learning approaches for this problem is given in [1]. In our case, inliers (or normal data) are phase fields containing stable compounds, and outliers (or anomalous data) are the phase fields without them. We assume that the outliers are not available to us at all and that we need to rely only on inliers to build a model, bringing us into the unsupervised learning setting. As described in the Section Machine learning models, we select an autoencoder for this task from a range of potential machine learning approaches.

Autoencoders are a class of non-linear dimensionality reduction techniques that are used in an unsupervised manner. An autoencoder trained on normal data should fail to reconstruct the anomalous data samples and produce large reconstruction errors for them. In addition, the variational autoencoder learns a probability distribution from the input normal data, improving its description. We use reconstruction errors to rank unseen phase fields by their closeness to the normal data.

In order to avoid overfitting, we employ the following strategy discussed in detail in the Section Model Validation. We hold-out a portion of our normal data prior to training and compare the distributions of reconstruction errors on the training and hold-out sets after fitting the model. Generalizability is deemed to be good when the resulting distributions are very similar to each other. In our case, we compare a simple statistic of these reconstruction errors. In general, validation of anomaly detection methods without access to a small sample of outliers is a subject of ongoing research[2].

In our case, we prefer models with a relatively broad distribution of reconstruction errors, when both average reconstruction errors and validation metrics are comparable. We expect such models to produce a ranking that is more discriminative and stable.

Training data and its representation are discussed in the Section Training set for the Variational Autoencoder Configuration.

Machine learning models

We studied different computationally undemanding machine learning methods[3] for phase field ranking. Each of these methods provides a method-specific metric of dissimilarity between the sample and normal data – the outlier score, which can be used to rank the phase fields. Since these algorithms are stochastic in nature, their outlier scores vary considerably depending on the initialization conditions, e.g., random seed number. In order to alleviate that, we take the mean value of scores across multiple runs as the final score, which requires a large number of iterations to achieve convergence (Supplementary Fig. 1). Noting that the more computationally involved neural-network-based approaches account for statistical variations by considering multiple models in a large network and a dropout[4], yielding converged results in every iteration, we employ a neural-network-based Variational Autoencoder (VAE), which uses the autoencoder's reconstruction error as a metric, in our study.

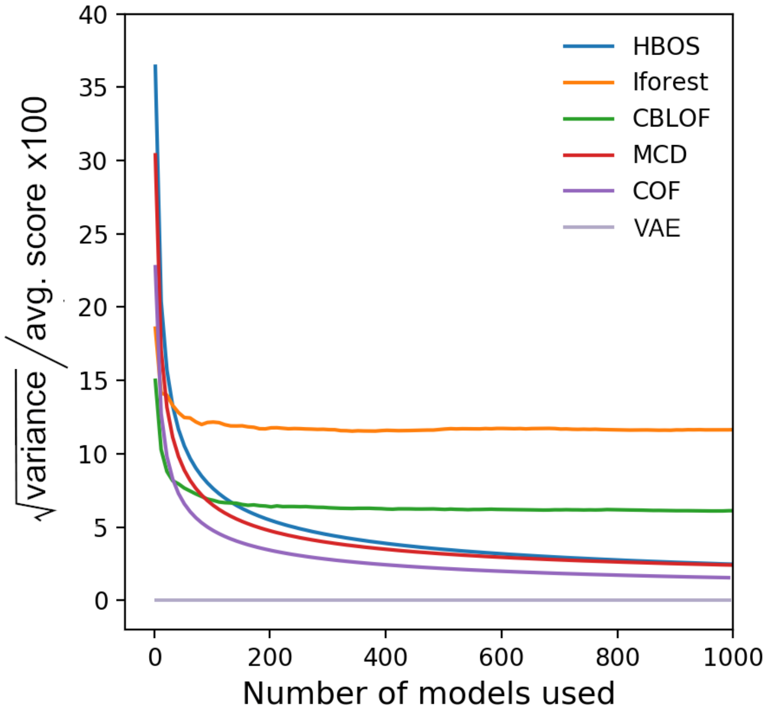

Supplementary Figure 1. Convergence of the different methods′ scores with the number of models used. ML methods used for phase field ranking are histogram-based (HBOS), isolation forest (Iforest), cluster-based (CBLOF), minimum covariance (MCD), connectivity-based (COD)[3]. Unlike the Variational Autoencoder neural network, accounting for statistical variations, up to 1000 iterations (models) are required to achieve convergence with these approaches.

Training set for the Variational Autoencoder Configuration

The open-source software (implementation of VAE and Phase Field Ranking method) developed for this study is available at https://www.github.com/lrcfmd/PhaseFieldRanking.

The training set contains all quaternary M-M′-A-A′ phase fields containing the $M_x M'_y A_z A'_t$ phases reported in ICSD-v1 2017 where:

- *M*, *M′* span all cations: all species of the periodic table with positive oxidation states
- *A*, *A′* are anions {N^{3-}, P^{3-}, As^{3-}, Sb^{3-}, O^{2-}, S^{2-}, Se^{2-}, Te^{2-}, F^-, Cl^-, Br^-, I^-}
- *x, y, z, t* are positive values and stand for stoichiometric coefficients
- There are reported phases in ICSD, where both M (positive oxidation state) and A

(negative oxidation state) are represented by the same element, e.g., phases containing both S^{6+}, S^{2-} . Such phases are not used in the training set.

The training process, in which reconstruction error (RE) and Kullback-Leibler (KL) divergence[5] of the probability distribution - the two components of the VAE loss function - are minimized, depends on the size of the dataset: for the original dataset (2021 entries) the neural network is overparametrized and requires many computationally expensive epochs of training to converge the loss function with “Adam” optimization[6]. Representation of a phase field in the elemental vector space (M, M', A, A') enables us to augment the training data 24-fold by elemental permutations. Indeed, 24 different vector representations, e.g., (M, M', A, A'), (A', M, M', A), (A, A', M, M') etc., are mathematically inequivalent, yet denote the same phase field. This enhances machine learning of the latent vector representation by improving the ratio of parameters to the number of entries, alleviating the risk of overfitting and decreasing the number of epochs required for convergence (Supplementary Fig. 2). Elemental permutations reflect that phase fields are sets that are order-invariant, allowing additional verification of the model that is required to produce identical results for permutations.

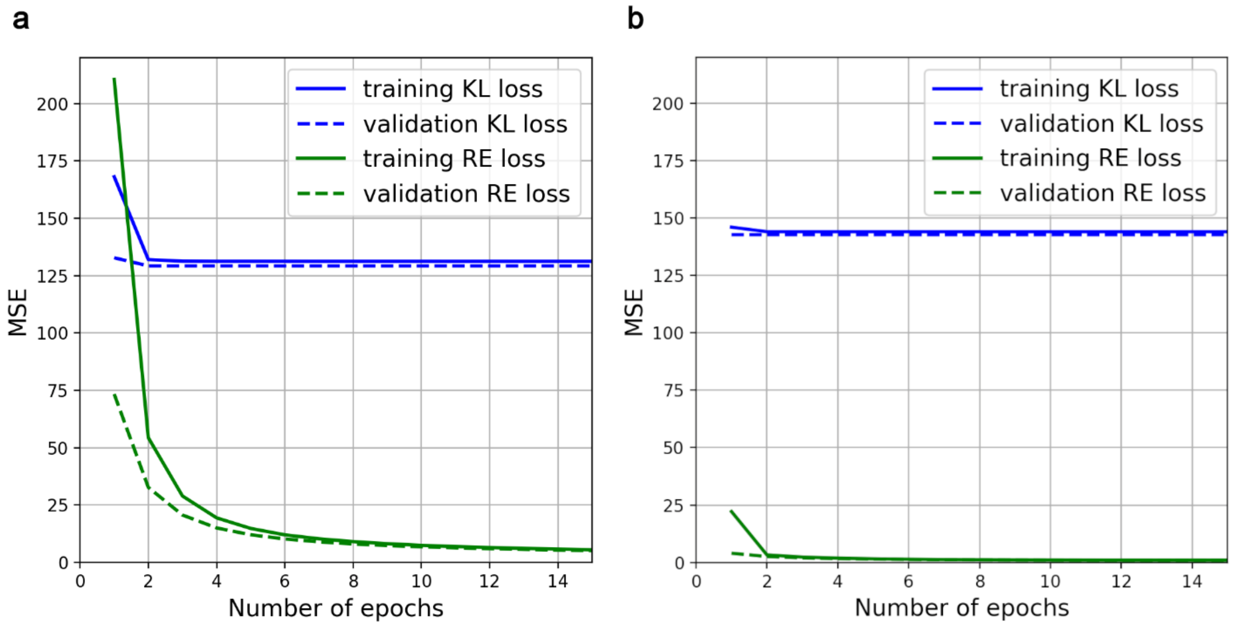

Supplementary Figure 2. VAE training mean square error (MSE) vs epoch for the original and augmented data. a Reconstruction error (RE) loss and Kullback-Leibler (KL) divergence loss in training of VAE on the augmented dataset via elemental permutations (48744 data points). Both losses converge within 15 epochs. b RE and KL divergence losses in training of VAE on 2021 data points. With the smaller training dataset, RE converges to the value in (a) (MSE = 0.976) in over 200 epochs, KL does not converge and oscillates with a small variance (MSE ~0.02).

In this paper, we use the VAE to learn the similarities between unexplored phase fields and the reported phase fields in ICSD. This is conducted by learning the multi-dimensional representations of the phase fields and their hidden features in the latent space with a dimensionality of choice in the way that best reconstructs the training data (RE and KL divergence are minimized). There is a small difference in distributions of RE between 2- and 4-dimensional latent space, and a robust distribution of RE that persists in choices of 4, 8, 16 dimensions for the latent space[7]. Thus, we choose 4-dimensional latent space for the model used.

Model validation

We perform 5-fold cross validation. In each of the five iterations, we hold out a different 20% of

the training data and train the model on the remaining 80%. We then apply the VAE model to the validation data (the separate 20%), and check that the distribution of scores of the validation set follows the distribution of the training set, ensuring the width, position and distribution of RE around the peak are within the same range – broadly analogous to the cohesion and separation methods for internal validation of unsupervised learning[8].

To simplify the comparison of RE for different data points, we use the normalised RE, calculated as $(RE - RE_{min})/(RE_{max} - RE_{min})$. The validation error is calculated as the percentage of the validation set that is distributed in the second half of the training distribution (i.e., the number of phase fields from the validation set which have the normalised RE higher than the threshold value of 0.5). The validation errors for different subsets (the separate 20%) of data chosen for validation are: 24.3, 20.1, 19.8, 26.2, 13.4%, demonstrating a comparable performance of the VAE to the state-of-the-art unsupervised machine learning techniques for various datasets[2,9,10]. The average validation error across the subsets is 20.76%, demonstrating an expected distribution of the RE in comparison to the training dataset.

Comparison of the distributions in the datasets of different sizes (in our case, ICSD training data - 2021 entries, testing data – 303 entries) can be addressed by methods in statistics for nonparametric density estimation[11], where the bin size, *w*, depends on the number of data entries, *N*, as follows:

$$w = \frac{2 \times IQR}{\sqrt[3]{N}} \qquad \text{(Supplementary Equation 1)}$$

where *IQR* is the interquartile range. These data size-adapted bins, with widths 0.04 for training and 0.077 for testing data respectively, allow size-independent comparison of RE distribution in the

training and testing data (Figure 2a in the main text and Supplementary Fig. 3). Such data distribution results in ten data entries located in the first bin of testing data.

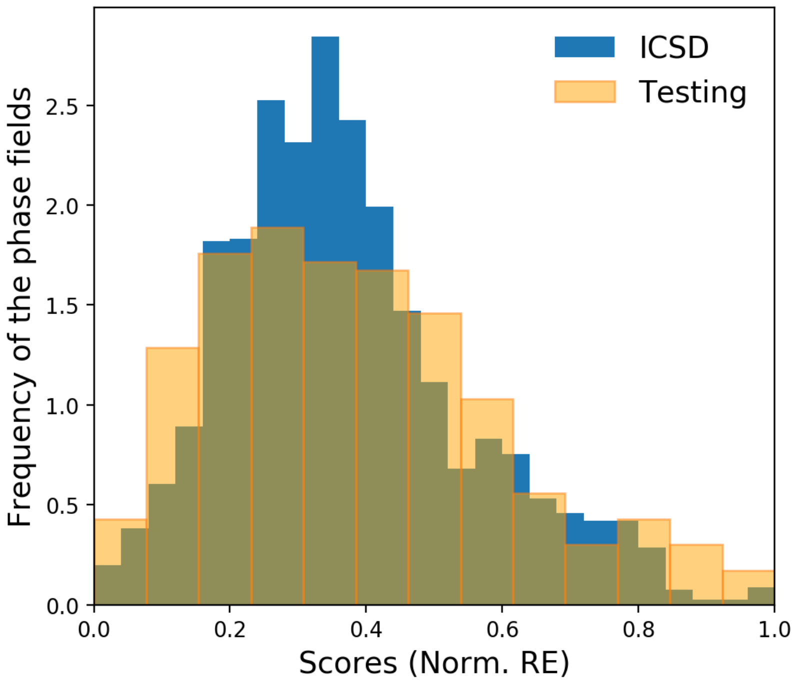

Supplementary Figure 3. A histogram of normalised RE (scores) in ICSD and testing datasets. The width of the bins depends on the number of entries in the two datasets, according to Supplementary Equation 1. The scores distributions demonstrate that the patterns for the majority of the data entries are well-recognised by the model (normalised RE < 0.5) with a few outliers.

Feature space

We study different basis sets of features for description of atomic elements built from 39 elemental features[12,13] (source https://bitbucket.org/wolverton/magpie/src/master/) plus the modified Pettifor number[14] of each element. Among the multitude of elemental features in the literature, we choose this set because the values are reported for all of the considered chemical elements, ensuring that there are no missing values in Supplementary Table 1.

Supplementary Table 1. Elemental features - values of atomic characteristics selected from[12–14]

atomic number	bcc band gap	ground state (GS) band gap	number of unfilled d-orbitals
atomic volume	bcc effective lattice constant	GS effective lattice constant	number of valence electrons in d-orbitals
atomic weight	bcc energy difference	GS energy per atom	number of unfilled f-orbitals
first ionization radius	bcc energy per atom	GS estimated bcc lattice constant	number of valence electrons in f-orbitals
covalent radius	bcc magnetic moment	GS estimated fcc lattice constant	number of unfilled p-orbitals
density	bcc volume per atom	GS magnetic moment	number of valence electrons in p-orbitals
electronegativity	bcc volume per atom difference	GS volume per atom	number of unfilled s-orbitals
boiling temperature	bcc Fermi energy	ICSD volume	number of valence electrons in s-orbitals
melting temperature	Periodic Table Group (column)	Periodic Table Period (row)	total number of unfilled orbitals
polarizability	number on the modified Pettifor scale.[14]	total number of valence electrons	Mendeleev number

As different combinations of features may have different efficiencies for phase field description

and resulting precision of the model, we study individual features' contributions to the reconstruction error of the phase fields in ICSD as well as the feature reconstruction error (FRE) - the relative change between the original and the reconstructed values of a feature averaged over all the phase fields that each element takes part in. For all the studied combinations of features discussed in this and the next paragraphs, we build a series of VAE models and examine their performance in terms of the validation error and the width of the score distributions. We begin with a set of all 40 features. In Supplementary Fig. 4 the average FRE is demonstrated for each element, enabling quantitative comparison of the effects of different features on the model's accuracy.

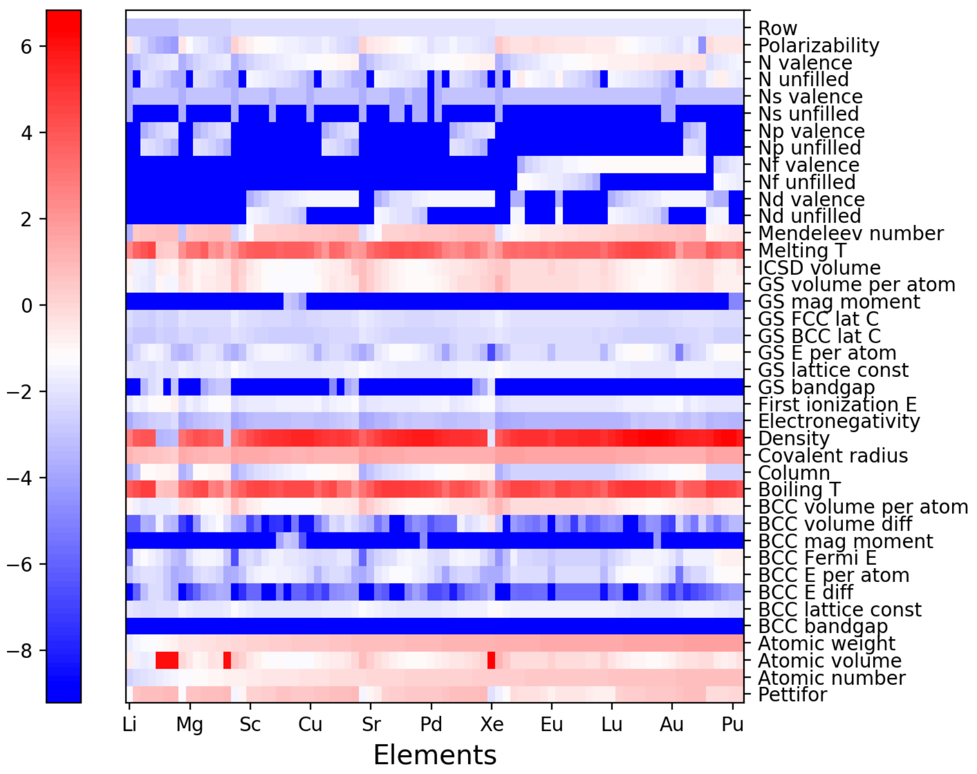

Supplementary Figure 4. Comparison of elemental features via feature reconstruction errors (FRE). FRE averaged for all atoms. 40 features are studied, and 3 features with high FRE (melting temperature, boiling temperature and density) are removed for the final model.

In the next model, we discard the most poorly reconstructed features (boiling temperature, melting temperature and density) to arrive at the basis of 37 features. In this basis, the phase fields are represented by concatenation of all the elemental descriptors of constituent atoms into 148-dimensional vectors. We also compare correlation (Pearson's correlation) of FRE of different features in Supplementary Fig. 5a. This reveals some strongly positively and negatively correlated pairs of atomic descriptors, including some well-known relations (*e.g.,* density and atomic weight, reverse correlations between covalent radius and first ionisation energy, polarisability and electronegativity[15], *etc.*). Supplementary Fig. 5b, however, demonstrates that FRE practically do not correlate with the total RE, suggesting the importance of maximizing the number of well-reconstructed elemental descriptors for detailed representation of the phase fields. We also confirm that in general, maximization of the number of features enhances the dispersion of the total RE between the data points in the model by increasing the width of the RE distribution, thereby improving the precision in discrimination between the phase fields and identification of the best candidates (Supplementary Fig. 5c).

We validate this elemental feature basis set by developing a Monte-Carlo (MC) approach, in which at every step a basis set of features is augmented or decreased with a randomly selected feature – a move that can be accepted or discarded depending on the VAE validation error produced with this basis (Supplementary Fig. 5d). The MC-derived basis set produces outcomes that are similar to but less effective than the set above (larger validation error, smaller distribution width), supporting the use of the set discussed above in the model.

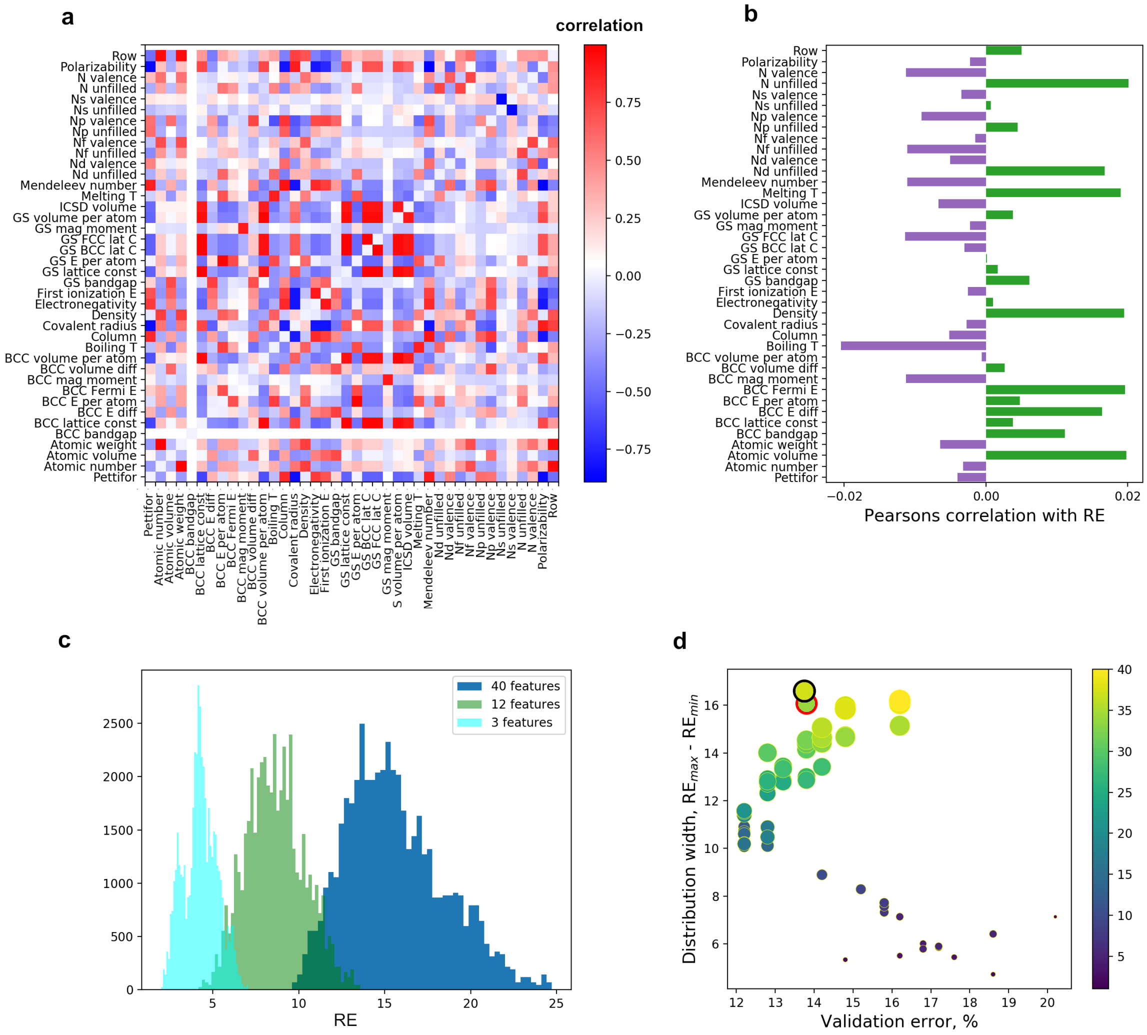

Supplementary Figure 5. Feature correlation, total RE and model precision and dispersion. **a** Pearson's correlation between pairs of individual feature reconstruction errors (FRE); **b** Averaged Pearson's correlation between individual FRE and total RE for quaternary compounds in ICSD, suggesting a strong non-linear dependence; **c** An increasing width of the distributions of RE for quaternaries in ICSD, when described with 3, 12, 40 elemental features improves discrimination between the RE of similar phase fields; **d** Different sets of elemental features representing quaternary phase fields found in the Monte-Carlo (MC) approach: the size and the colour of a circle corresponds to the number of elemental features in a set, its location on the x-axis corresponds to the resulting VAE validation error, when quaternary phase fields in ICSD are described with this set of features, while the y-axis corresponds to the width of the corresponding RE distribution attainable with this set. The best compromise between the range of RE and a low validation error found with MC is the set of 34 features (circled in red); the set of 37 features selected for the final model used is circled in black.

Synthesis

Samples with composition $LiSnS_{2.33}Cl_{0.33}$ (#1), Li_3SnS_3Cl (#2), $Li_{1.5}SnS_{1.5}Cl_{2.5}$ (#3), $Li_8SnS_4Cl_4$ (#4), $Li_{1.67}SnS_{0.33}Cl_5$ (#5), $Li_{11}SnS_3Cl_9$ (#6), $Li_{3.6}SnS_{3.6}Cl_{0.4}$ as well as the purified $Li_{3.3}SnS_{3.3}Cl_{0.7}$

new phase were all synthesized by solid state reaction in alumina crucibles contained in evacuated sealed quartz tubes using the same procedure as described in the method section of the main text. Stoichiometric amounts of Li_2S (Merck, 99.98 %), SnS (Alfa Aesar, 99+ %), LiCl (Merck, 99.99 %), S (Merck, 99.98 %), $SnCl_2$ (Merck, >99.99 %) and $SnCl_4$ (Merck, 99.995 %) precursors were weighed in order to yield a total mass of powder of approximately 300 mg (the exact amount of precursors for each sample are given in Supplementary Table 2). Powders were combined and mixed thoroughly with an agate pestle and mortar for 15 min (except for the $SnCl_4$ containing sample for which powder precursor were simply poured into liquid $SnCl_4$), transferred into an alumina crucible and then sealed in a quartz tube under a pressure of 10^{-4} mbar. The ampoule containing the sample was heated to 700 °C at a ramp rate of 5 °C min^{-1}, held at 700 °C for 12 hours, and then cooled to room temperature at a ramp rate of 5 °C min^{-1}. The resulting powder was then manually ground in order to obtain a fine powder. Precursors and resulting powders were handled in an Ar-filled glovebox (O_2 < 1 ppm).

Supplementary Table 2. Quantities of precursors weighed for each sample made in the Li-Sn-S-Cl phase field.

Sample code	Composition	Precursor weight (mg)	
#1	$LiSnS_{2.33}Cl_{0.33}$	Li_2S	23
		SnS	226.2
		S	48.1
		LiCl	21.2
#2	Li_3SnS_3Cl	Li_2S	51.7
		SnS	169.6
		S	36.1
		LiCl	47.7
#3	$Li_{1.5}SnS_{1.5}Cl_{2.5}$	$SnCl_2$	107
		SnS	85.1
		S	36.2
		LiCl	71.8
#4	$Li_8SnS_4Cl_4$	Li_2S	70.3
		SnS	115.3
		S	24.5
		LiCl	129.7
#5	$Li_{1.67}SnS_{0.33}Cl_5$	$SnCl_4$	205.2

		SnS	23.5
		S	5
		LiCl	66.4
#6	$Li_{11}SnS_3Cl_9$	Li_2S	23
		SnS	75.4
		S	16
		LiCl	190.8
#7	$Li_{3.6}SnS_{3.6}Cl_{0.4}$	Li_2S	85.5
		SnS	175.3
		S	37.3
		LiCl	19.7
Phase A	$Li_{3.3}SnS_{3.3}Cl_{0.7}$	Li_2S	73.5
		SnS	180.9
		S	38.5
		LiCl	33.9

Elemental analysis

Inductively Coupled Plasma Atomic Emission Spectroscopy (ICP-AES). Elemental analysis of $Li_{3.3}SnS_{3.3}Cl_{0.7}$ was performed by Mikroanalytisches Labor Pascher at Remagen-Bandorf, Germany, to determine Li, Sn, S and Cl content. Samples were handled under inert atmosphere.

WDX analysis. Wavelength dispersive X-ray spectroscopy (WDX) measurements were performed in order to determine the Cl content and further validate Sn and S contents. This was done using a TESCAN S8000 scanning electron microscope (SEM) equipped with a WAVE WDX detector from Oxford Instruments. A pellet of $Li_{3.3}SnS_{3.3}Cl_{0.7}$ was transferred from an argon filled glovebox to the instrument using a vacuum transfer holder from Quorum. The pellet was coated with a thin layer of platinum with a Quorum sputter coater in order to minimize the effects of charging. Measurements and quantifications were performed using the software INCA from Oxford Instruments. For accurate quantification, data were also collected from standard materials (SnO_2, Bi_2S_3, NaCl standards supplied by the manufacturer) for each element.

Diffraction and refinement

Routine analysis of phase purity and lattice parameters were performed on a Bruker D8 Advance diffractometer with a monochromated Cu source ($K\alpha_1$, $\lambda = 1.54060$ Å) in powder transmission Debye Scherrer geometry (capillary) with sample rotation.

The structural models were refined by the Rietveld method as implemented in the Fullprof suite[16]. For the sake of realism, all uncertainties were increased by Berar's factor (4.5, 3.2 and 3.9 for SXRD, NPD Bank 4 and NPD Bank 5, respectively according to FullProf).

The program FullProf was used to obtain information about the microstructure, following the method described by Rodriguez-Carvajal *et al*[16,17]. This model uses the Scherrer formula, which considers that the size broadening can be written as a linear combination of spherical harmonics. Peak shapes were modelled using the spherical harmonics expansions in a hexagonal material with Laue class 6/*mmm*.

Maximum Entropy Method Analysis of Diffraction Data

The maximum entropy method (MEM) applied to diffraction data consists of optimizing the reconstruction of the scattering density from the observed structure factors by finding the maximum of the informational entropy under several constraints through an iterative procedure[18]. MEM applied to crystallography is a powerful tool for reconstructing scattering density from incomplete and/or noisy data systems and limits termination effects obtained through usual Fourier synthesis, particularly important in disordered systems[19]. The maximum entropy method (MEM) applied to neutron diffraction data is useful to shed light on the positions of light elements, such as Li, poorly visible with X-rays, but presenting large enough neutron scattering length. This method was recently used to describe conduction pathways in several ionic conductors of lithium[20,21] and

oxygen[22] in particular.

Because the ^{7}Li scattering length is negative (b_{Li} = -2.22 fm), visualisation of negative levels is performed to observe Li positions within the structure. The background level was set as the maximum value of the nuclear density obtained in regions where no atoms are expected (surroundings of the S site): 0.001 fm Å^{-3}.

DC polarisation measurements

For DC polarization measurements, a cylindrical pellet of $Li_{3.3}SnS_{3.3}Cl_{0.7}$ with a thickness of 1.75(9) mm and a surface area of 0.18(9) cm^2 was used. An Au|$Li_{3.3}SnS_{3.3}Cl_{0.7}$|Au ion-blocking electrode was subjected to different DC bias voltages (0.1 to 1V) at room temperature. The current was recorded as a function of time. The steady state current corresponds to the electronic current. Prior to the DC polarization, the AC impedance was collected from 3 MHz to 1 mHz with a voltage amplitude of 100 mV using a Biologic VSP-300 potentiostat/galvanostat.

Electrochemical Li plating/stripping

Symmetrical Li|$Li_{3.3}SnS_{3.3}Cl_{0.7}$|Li cells were assembled inside an Ar-filled glovebox (O_2, H_2O ≤ 0.1 ppm). Two-electrode Swagelok cells were used with PEEK-insulated body and uniform spring-loaded compression (Micro Plas Mouldings Ltd.). Lithium ribbon (99.9%, 0.38 mm thick, Sigma) was scraped to remove surface layers, then finely polished to achieve a shiny mirrored surface and punched into discs. $Li_{3.3}SnS_{3.3}Cl_{0.7}$ pellet (*ca*. 1.5 mm thickness) was sandwiched between two pieces of Li discs and carefully aligned inside the cell. The cell was sealed hermetically under compression before being transferred to a temperature chamber (Memmert). Li plating/stripping was measured by galvanostatic cycling at 0.01 mA cm^{-2} at 303, 323 and 343 K for 1 h per half-cycle

using an MPG2 workstation (Biologic). The cell was allowed to thermally equilibrate for a minimum of 1 h between the temperature steps. EIS measurements were performed in 50 cycle intervals using an SP-300 potentiostat (Biologic) with a 20 mV sinusoidal perturbation between 7 MHz and 100 mHz at open circuit potential.

Ex-situ Raman microscopy and XRD after Li plating/stripping

After completing Li plating/stripping measurements, the symmetric cell was disassembled inside the Ar-filled glovebox. The pellet material was collected and sealed inside a custom-built stainless-steel holder with a CaF_2 window. Raman spectra of the cycled and pristine solid electrolyte were collected using a Renishaw In-Via Rama spectrometer equipped with an inverted microscope to provide spatial sensitivity and the ability to focus on individual particles. Spectra were collected with 785 nm wavelength laser at room temperature. Laboratory XRD patterns were collected for material extracted from the bulk of the cycled pellet, and powder scraped from the Li|$Li_{3.3}SnS_{3.3}Cl_{0.7}$ interfacial region. This interfacial powder was mixed with amorphous boron and sealed in a 0.5 mm diameter borosilicate capillary for measurement.

Nuclear Magnetic Resonance (NMR)

SLR rates in the laboratory frame (T_1^{-1}) were obtained using a saturation recovery pulse sequence and the data were fitted to a stretch exponential function of the form:

$$1 - exp[-(\tau/ T_1^{-1})^{\alpha}] \quad \text{(Supplementary Equation 2)}$$

where τ are the variable delays and α is the stretch exponential (values between 0.4 and 1).

SLR rates in the rotating frame ($T_{1\rho}^{-1}$) were recorded using a standard spin-lock pulse sequence at frequencies of $\omega_1/2\pi$ (^{7}Li) = 15, 45, and 80 kHz, and data were fitted to a stretch exponential

function of the form:

$$exp[-(\tau/T_{1\rho}^{-1})^{\beta}] \quad \text{(Supplementary Equation 3)}$$

where β values are between 0.3 and 1.

Temperature calibrations were performed using the chemical shift thermometers $Pb(NO_3)_2$, CuI and CuBr using ^{207}Pb and ^{63}Cu NMR.[23–26] The errors associated with this method were calculated using the broadening of the isotropic peak and ranged from 5 - 20 K.

Supplementary Discussion

Ranking of the quaternary unexplored Li-*M*-*A*-*A′* phase fields

For the ranking, we calculated reconstruction errors (RE) of all phase fields of interest as the Euclidean distance between an original 148-dimensional vector, p, and its 148-dimensional image vector, p′, decoded by the VAE, presented in Supplementary Table 3. For the ease of comparison, we also normalise the RE (Norm. RE) by scaling them with the width of the distribution ($RE_{max} - RE_{min}$) and presenting all scores from 0 to 1.

Supplementary Table 3. Ranking of 303 Li-M-A-A′ phase fields.

Phase fields	RE	Norm. RE	Phase fields	RE	Norm. RE	Phase fields	RE	Norm. RE
Li Si S Cl	9.782	0	Li Zr S I	11.402	0.269	Li Zr N O	12.481	0.448
Li Zn S Cl	9.931	0.025	Li Al O F	11.402	0.269	Li Ta S I	12.513	0.453
Li Al S Cl	10.041	0.043	Li Mg N Cl	11.408	0.27	Li Ta I Cl	12.57	0.463
Li Mg S Cl	10.05	0.044	Li Mg O F	11.41	0.27	Li Sr O Br	12.573	0.463
Li Sn S Cl	10.095	0.052	Li Ca Br Cl	11.424	0.272	Li La O Br	12.574	0.463
Li Si S Br	10.115	0.055	Li N P Br	11.441	0.275	Li Ca N Br	12.579	0.464

Li P Br Cl	10.158	0.062	Li Ca S O	11.442	0.275	Li Zr N I	12.615	0.47
Li P S O	10.177	0.066	Li B O Br	11.447	0.276	Li Y Br F	12.619	0.471
Li Si Br Cl	10.185	0.067	Li Sn N Cl	11.448	0.276	Li Y N S	12.634	0.473
Li Si S O	10.205	0.07	Li Sn O F	11.45	0.277	Li Ca N O	12.652	0.476
Li Zn S Br	10.259	0.079	Li Zn I F	11.453	0.277	Li Sr S F	12.684	0.481
Li Si O Cl	10.275	0.082	Li Zr I Cl	11.464	0.279	Li La S F	12.685	0.482
Li Zn Br Cl	10.328	0.091	Li Si N Br	11.465	0.279	Li Y N Cl	12.69	0.482
Li Zn S O	10.348	0.094	Li Y S Cl	11.485	0.282	Li Y O F	12.692	0.483
Li Al S Br	10.365	0.097	Li Ca O Cl	11.504	0.286	Li Ta O Br	12.7	0.484
Li Si S I	10.368	0.097	Li Al I F	11.548	0.293	Li Sr I Br	12.706	0.485
Li Mg S Br	10.374	0.098	Li Mg I F	11.556	0.294	Li La I Br	12.707	0.485
Li P I Cl	10.41	0.104	Li B S F	11.568	0.296	Li Sr Cl F	12.74	0.491
Li Zn O Cl	10.417	0.105	Li Ca S I	11.588	0.299	Li La Cl F	12.741	0.491
Li Sn S Br	10.418	0.105	Li B I Br	11.592	0.3	Li B N F	12.766	0.495
Li Al Br Cl	10.434	0.108	Li Zn N Br	11.593	0.3	Li Sr O I	12.778	0.497
Li Si I Cl	10.437	0.109	Li Sn I F	11.596	0.301	Li La O I	12.779	0.497
Li Mg Br Cl	10.443	0.11	Li Zr O Br	11.606	0.302	Li Ca N I	12.784	0.498
Li Al S O	10.453	0.111	Li B Cl F	11.63	0.307	Li Ta S F	12.809	0.502
Li Mg S O	10.462	0.113	Li Ca I Cl	11.649	0.31	Li Y I F	12.823	0.504
Li Sn Br Cl	10.486	0.117	Li N P I	11.665	0.312	Li Ta I Br	12.831	0.506
Li Sn S O	10.506	0.12	Li B O I	11.671	0.313	Li Ta Cl F	12.865	0.511
Li Zn S I	10.509	0.121	Li Zn N O	11.671	0.313	Li Ba S Cl	12.902	0.517
Li Al O Cl	10.521	0.123	Li Al N Br	11.687	0.316	Li Ta O I	12.902	0.518
Li Mg O Cl	10.53	0.124	Li Si N I	11.69	0.316	Li Zr N F	12.909	0.519
Li Sn O Cl	10.573	0.131	Li Mg N Br	11.695	0.317	Li Y N Br	12.948	0.525
Li Zn I Cl	10.577	0.132	Li Zr S F	11.726	0.322	Li Sr Br F	12.998	0.533
Li Si O Br	10.592	0.134	Li Sn N Br	11.734	0.324	Li La Br F	12.998	0.534

Li Al S I	10.612	0.138	Li Zr I Br	11.749	0.326	Li Sr N S	13.011	0.536
Li Mg S I	10.621	0.139	Li Al N O	11.765	0.329	Li La N S	13.012	0.536
Li Sn S I	10.664	0.146	Li Y S Br	11.769	0.33	Li Y N O	13.019	0.537
Li Al I Cl	10.68	0.149	Li Mg N O	11.773	0.33	Li Sr N Cl	13.066	0.545
Li Mg I Cl	10.688	0.15	Li Zr Cl F	11.787	0.332	Li La N Cl	13.067	0.545
Li P S F	10.697	0.152	Li Ca O Br	11.789	0.333	Li Sr O F	13.068	0.545
Li B S Cl	10.702	0.153	Li Sn N O	11.812	0.337	Li La O F	13.069	0.545
Li P I Br	10.723	0.156	Li Zn N I	11.814	0.337	Li Ca N F	13.073	0.546
Li Si S F	10.724	0.156	Li Zr O I	11.827	0.339	Li Ta Br F	13.12	0.554
Li Zn O Br	10.73	0.157	Li Y Br Cl	11.83	0.34	Li Ta N S	13.134	0.556
Li Sn I Cl	10.731	0.157	Li Y S O	11.847	0.342	Li Y N I	13.147	0.558
Li Si I Br	10.749	0.16	Li Sr S Cl	11.899	0.351	Li Ba S Br	13.156	0.56
Li P Cl F	10.764	0.163	Li La S Cl	11.9	0.351	Li Ta N Cl	13.188	0.565
Li Si Cl F	10.79	0.167	Li Al N I	11.907	0.352	Li Sr I F	13.196	0.566
Li P O I	10.808	0.17	Li Ca S F	11.907	0.352	Li La I F	13.197	0.566
Li Al O Br	10.831	0.174	Li Y O Cl	11.907	0.352	Li Ba Br Cl	13.21	0.569
Li Si O I	10.834	0.175	Li B Br F	11.911	0.353	Li Ba S O	13.225	0.571
Li Mg O Br	10.84	0.175	Li Mg N I	11.915	0.354	Li Ba O Cl	13.279	0.58
Li Zn S F	10.859	0.179	Li B N S	11.926	0.356	Li Ta I F	13.316	0.586
Li Zr S Cl	10.871	0.181	Li Ca I Br	11.93	0.356	Li Sr N Br	13.317	0.586
Li Sn O Br	10.882	0.182	Li Sn N I	11.953	0.36	Li La N Br	13.318	0.587
Li Zn I Br	10.885	0.183	Li Ca Cl F	11.967	0.362	Li Ba S I	13.352	0.592
Li Zn Cl F	10.925	0.19	Li N P F	11.982	0.365	Li Sr N O	13.386	0.598
Li Al S F	10.96	0.195	Li B N Cl	11.986	0.366	Li La N O	13.387	0.598
Li Mg S F	10.968	0.197	Li Y S I	11.988	0.366	Li Ba I Cl	13.405	0.601
Li Zn O I	10.969	0.197	Li Si N F	12.006	0.369	Li Y N F	13.429	0.605
Li Al I Br	10.985	0.2	Li Ca O I	12.007	0.369	Li Ta N Br	13.437	0.606

Li Mg I Br	10.994	0.201	Li Ta S Cl	12.032	0.373	Li Sr N I	13.511	0.618
Li B S Br	11.006	0.203	Li Y I Cl	12.047	0.376	Li La N I	13.511	0.619
Li Sn S F	11.01	0.204	Li Zr Br F	12.064	0.379	Li Ba O Br	13.526	0.621
Li Al Cl F	11.025	0.206	Li Zr N S	12.079	0.381	Li Ta N I	13.628	0.638
Li Mg Cl F	11.033	0.208	Li B I F	12.127	0.389	Li Ba S F	13.629	0.638
Li Sn I Br	11.035	0.208	Li Zn N F	12.127	0.389	Li Ba I Br	13.65	0.642
Li Ca S Cl	11.066	0.213	Li Zr O F	12.14	0.391	Li Ba Cl F	13.682	0.647
Li P Br F	11.067	0.213	Li Sr S Br	12.174	0.397	Li Ba O I	13.717	0.653
Li Al O I	11.068	0.213	Li La S Br	12.175	0.397	Li Sr N F	13.785	0.664
Li B Br Cl	11.071	0.214	Li Y O Br	12.182	0.398	Li La N F	13.786	0.664
Li Sn Cl F	11.075	0.214	Li Al N F	12.217	0.404	Li Ta N F	13.901	0.683
Li Mg O I	11.077	0.215	Li Mg N F	12.225	0.405	Li Ba Br F	13.922	0.687
Li N P S	11.083	0.216	Li Sr Br Cl	12.232	0.406	Li Ba N S	13.934	0.689
Li B S O	11.089	0.217	Li La Br Cl	12.233	0.407	Li Ba N Cl	13.986	0.697
Li Si Br F	11.093	0.217	Li Ca Br F	12.24	0.408	Li Ba O F	13.987	0.698
Li Si N S	11.109	0.22	Li Sr S O	12.249	0.409	Li Ba I F	14.107	0.717
Li Sn O I	11.118	0.222	Li La S O	12.25	0.409	K Li S Cl	14.196	0.732
Li N P Cl	11.148	0.227	Li Ca N S	12.255	0.41	Li Ba N Br	14.22	0.736
Li P O F	11.15	0.227	Li B N Br	12.259	0.411	Li Ba N O	14.285	0.747
Li Zr S Br	11.172	0.23	Li Sn N F	12.262	0.411	Li Ba N I	14.402	0.766
Li Si N Cl	11.173	0.231	Li Zr I F	12.277	0.414	K Li S Br	14.427	0.771
Li Si O F	11.175	0.231	Li Y S F	12.296	0.417	K Li Br Cl	14.477	0.779
Li Zn Br F	11.224	0.239	Li Ta S Br	12.304	0.418	K Li S O	14.491	0.781
Li Zr Br Cl	11.236	0.241	Li Sr O Cl	12.307	0.419	K Li O Cl	14.54	0.789
Li B S I	11.24	0.242	Li La O Cl	12.308	0.419	K Li S I	14.606	0.8
Li Zn N S	11.24	0.242	Li Ca N Cl	12.313	0.42	K Li I Cl	14.655	0.808
Li Zr S O	11.253	0.244	Li Ca O F	12.315	0.42	Li Ba N F	14.66	0.809

Li P I F	11.299	0.252	Li Y I Br	12.319	0.421	K Li O Br	14.766	0.827
Li B I Cl	11.303	0.252	Li B N O	12.334	0.423	K Li S F	14.861	0.842
Li Zn N Cl	11.304	0.252	Li Y Cl F	12.354	0.427	K Li I Br	14.879	0.845
Li Zn O F	11.305	0.253	Li Ta Br Cl	12.362	0.428	K Li Cl F	14.909	0.85
Li Zr O Cl	11.317	0.255	Li Ta S O	12.379	0.431	K Li O I	14.941	0.856
Li Al Br F	11.321	0.255	Li Sr S I	12.385	0.432	K Li Br F	15.129	0.887
Li Si I F	11.324	0.256	Li La S I	12.386	0.432	K Li N S	15.141	0.889
Li Mg Br F	11.329	0.257	Li Y O I	12.393	0.433	K Li N Cl	15.188	0.897
Li Al N S	11.337	0.258	Li Zr N Br	12.408	0.436	K Li O F	15.19	0.897
Li Mg N S	11.345	0.259	Li Ta O Cl	12.436	0.44	K Li I F	15.3	0.915
Li Ca S Br	11.362	0.262	Li Sr I Cl	12.443	0.441	K Li N Br	15.404	0.933
Li Sn Br F	11.37	0.263	Li La I Cl	12.444	0.441	K Li N O	15.464	0.942
Li Sn N S	11.385	0.266	Li Ca I F	12.45	0.443	K Li N I	15.572	0.96
Li Al N Cl	11.4	0.268	Li B N I	12.469	0.446	K Li N F	15.811	1

Supplementary Table 4. Reported ionic conductivities for ternary compounds in the top 5 quaternary phase fields in Supplementary Table 3.

Compound	Conductivity ($S \cdot cm^{-1}$)	Reference
Li_2SiS_3	2×10^{-6} (25°C)	Ahn *et al.*[27]
Li_4SiS_4	5×10^{-8} (25°C)	Ahn *et al.*[27]
Li_2ZnCl_4	8.1×10^{-6} (200°C)	Lutz *et al.*[28]
Li_2MgCl_4	$\sim 10^{-5}$ (25°C)	Kanno *et al.*[29]
Li_3AlS_3	1.3×10^{-8} (25°C)	Gamon *et al.*[30]
Li_5AlS_4	9.7×10^{-9} (25°C)	Lim *et al.*[31]
Li_4SnS_4 orthorhombic	7.0×10^{-5} (25°C)	Kaib *et al.*[32]
Li_4SnS_4 hexagonal	1.1×10^{-4} (25°C)	Kanazawa *et al.*[33]
$Li_{0.8}Sn_{0.8}S_2$	1.5×10^{-2} (25°C)	Holtzmann *et al.*[34]
Li_2SnS_3	1.5×10^{-5} (25°C)	Brant *et al.*[35]

Experimental phase diagram evaluation

Using the results from experiments #2 and #6, the composition of Phase A can be averaged considering the molar ratio of impurities present in each sample (#2: $Li_{0.8}Sn_{0.8}S_2$/LiCl = 0.9; #6: SnS_2/LiCl ~ 0.05), determined by the Rietveld method. The relative weight fractions of impurities derived from Rietveld refinement on these two samples were used to form a system of equations and determine the subsequent composition to make. The intersection of the two lines derived from equations: $Li_3SnS_3Cl - x \times (0.9 \times Li_{0.8}Sn_{0.8}S_2 + LiCl)$ and $Li_{11}SnS_3Cl_9 - x \times (0.05 \times SnS_2 + LiCl)$ (short and long yellow lines, respectively, on Figure 2b) gives a composition close to $Li_{3.6}SnS_{3.6}Cl_{0.4}$.

Supplementary Table 5. Compositions, sample code in Figure 2b (main text) and phases obtained after reaction in a sealed quartz tube at 700 °C for 12 hours identified by XRD.

Sample code	Composition	Phases identified by XRD
#1	$LiSnS_{2.33}Cl_{0.33}$	$Li_{0.8}SnS_2$<br>LiCl<br>Li_2SnS_3
#2	Li_3SnS_3Cl	Phase A<br>$Li_{0.8}Sn_{0.8}S_2$, LiCl<br>($Li_{0.8}Sn_{0.8}S_2$/LiCl = 0.9)
#3	$Li_{1.5}SnS_{1.5}Cl_{2.5}$	SnS_2<br>LiCl
#4	$Li_8SnS_4Cl_4$	Li_2S<br>LiCl<br>Li_4SnS_4
#5	$Li_{1.67}SnS_{0.33}Cl_5$	SnS_2<br>LiCl
#6	$Li_{11}SnS_3Cl_9$	Phase A<br>LiCl, SnS, S(am)<br>(SnS_2/LiCl= 0.05)

#7	$Li_{3.6}SnS_{3.6}Cl_{0.4}$	Phase A<br>Li_4SnS_4
Phase A	$Li_{3.3}SnS_{3.3}Cl_{0.7}$	Phase A

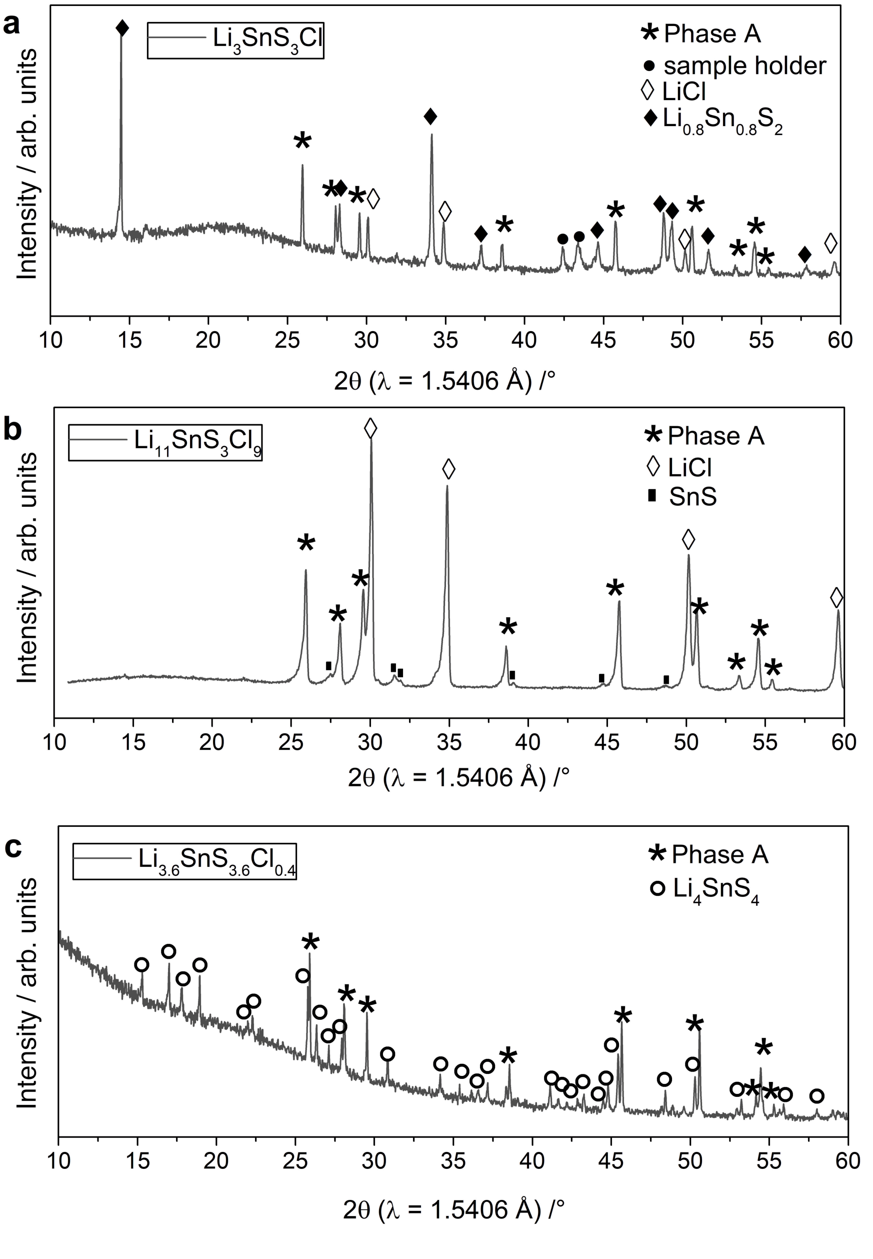

Supplementary Figure 6. Laboratory XRD patterns of samples. a #2, **b** #6 and **c** #7 made in the Li-Sn-S-Cl phase field (cf. Figure 2b and Supplementary Table 5), synthesized in sealed quartz tubes at 700 °C for 12 h. Known phases are denoted with signs defined in the legend. Phase A is denoted with the "*" sign.

Crystal structure determination

The observed reflections for the SXRD pattern of $Li_{3.3}SnS_{3.3}Cl_{0.7}$ indicated the presence of a 6_3 screw axis and a *c* glide plane, therefore leaving the possibility for the three space groups $P6_3mc$, $P\bar{6}2c$ and $P6_3/mmc$ (extinction symbol $P - - c$).[36] The presence of a minor impurity of orthorhombic Li_4SnS_4 was identified in the SXRD pattern and this phase was included in the refinement. The final refinement yields no more than 4 wt% of this phase.

A preliminary structural model based on the wurtzite structure (space group $P6_3mc$) in which S and Cl atoms share the anion site in a S/Cl = 3.3/0.7 ratio (site occupancy factor, *sof*, for S and Cl of 0.825 and 0.175, respectively), and where the cation site is partially occupied by Sn and Li, with *sof* of 0.25 and 0.75, respectively, was used. The remaining Li atoms were omitted in the first step of the refinement. After refining cationic and anionic positions and isotropic displacement parameters, a good fit against SXRD data was obtained, revealing appropriate positions for the heavier atoms Sn, S/Cl in the $Li_{3.3}SnS_{3.3}Cl_{0.7}$ structure.

The screening of the Fourier difference map using the NPD data, revealed the presence of a negative nuclear density in the octahedral interstice of the anion sublattice (Supplementary Fig. 7). A lithium atom was added in this position and its occupancy refined to 0.092(8) while the goodness of fit for the refinement, χ^2, decreased from 9.53 and 6.90 to 5.69 and 1.59 for Banks 4 and 5, respectively.

A Rietveld refinement using a structural model containing a Li atom on the T^- site was envisaged (constraining the overall occupancy of the T^+-T^- face shared unit to be less than 1). However, at such low occupancy, the error obtained on the refined *sof* was too high (*sof* = 0.01(2)) for this site to be kept in the final structural model. The position, displacement parameters and *sof* of all atoms were then refined simultaneously constraining the composition to be charge-neutral, with no constraint of full occupancy for the anion site, and this yielded the final model. The outcome of the

refinement is presented in Supplementary Table 6 and Supplementary Table 7.

The final refined composition is $Li_{3.41(4)}SnS_{3.29(3)}Cl_{0.70(3)}$, close to the measured composition $Li_{3.305(14)}Sn_{1.000(9)}S_{3.317(44)}Cl_{0.6269(8)}$ as determined by ICP-AES. Measurements from SEM-WDX (*cf.* Methods) show that the material is homogeneous and yields a measured elemental ratio of 1.000(6):3.57(4):0.61(4) for Sn:S:Cl which agrees well the expected values of 1:3.3:0.7 and confirming the assumption required for the multiple source refinement of anion occupancies that S and Cl are the only elements occupying the anion sites.

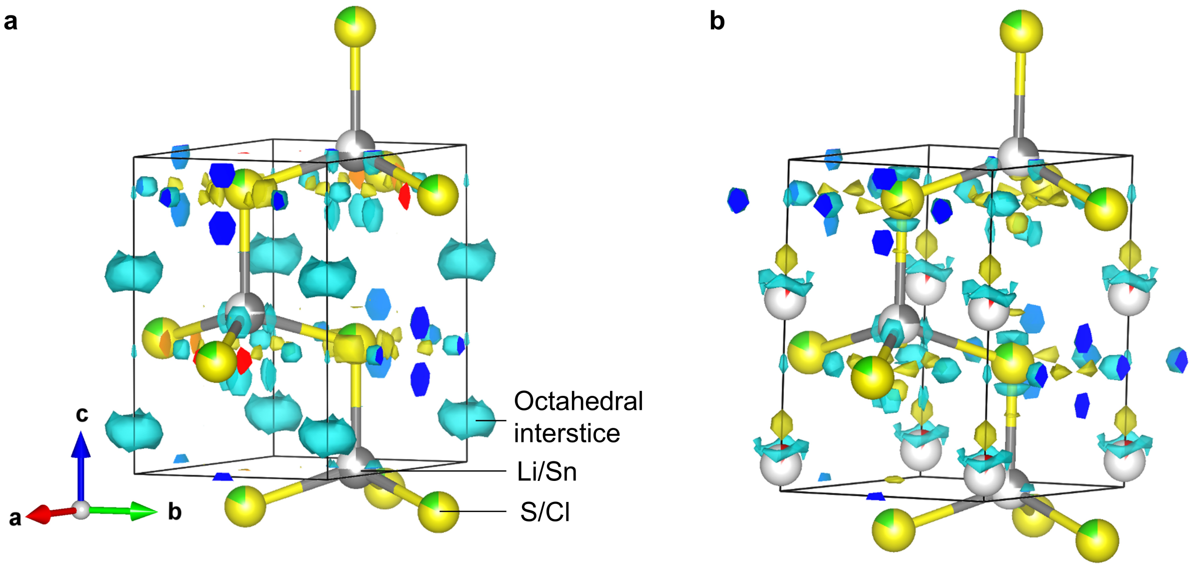

Supplementary Figure 7. Fourier difference map from the refinement of the NPD data from Bank 4 of Polaris using: **a** the wurtzite structural model for $Li_{3.3}SnS_{3.3}Cl_{0.7}$, showing the negative nuclear density at the octahedral site of the anion sublattice and **b** Fourier difference map after refining Li occupancy in the octahedral interstice.

Supplementary Table 6. Summary of the outcome of the refinement of $Li_{3.3}SnS_{3.3}Cl_{0.7}$ against synchrotron X-ray powder diffraction (SXRD) and neutron powder diffraction (NPD) data.

Radiation	NPD Bank 4	NPD Bank 5	SXRD
**Refined composition**	$Li_{3.41(4)}SnS_{3.29(3)}Cl_{0.70(3)}$		
**Formula weight (g·mol^{-1})**	272.69		
**Space group**	$P6_3mc$		
Z	2		
**Density (g·cm^{-3})**	2.570		
**Temperature (K)**	298		

**Angle (°) / Wavelength (Å)**	92.590	146.720	0.82637
***d* spacing range (Å)**	0.74043 - 3.66017	0.54095 - 2.56047	0.57379 - 22.61176
**TOF (µsec.) / 2*θ* (°) range**	1107.354858-19937.962891	1103.836304 - 19944.859375	2.084000 - 92.116005
**TOF (µsec.) / 2*θ* (°) step**	6.5135	3.2546	0.004000
**No. of reflections**	162	326	220
**No. of data**	2893	5791	22509
**No. of refined parameters**	16 (profile)<br>11 (atomic)		18 (profile)<br>11 (atomic)
***a* (Å)**	3.9723(3)		
***c* (Å)**	6.3524(6)		
**Volume (Å^3)**	87.06(2)		
R_p	9.36	10.8	17.4
R_{wp}	8.87	10.6	15.7
R_{exp}	3.72	8.42	2.00
R_{Bragg}	5.57	4.53	7.95
χ^2	5.69	1.59	61.8
$\rho_{min./max.}$ **residuals (fm/e$^-$·Å^{-3})**	-0.01 / 0.02	-0.02 / 0.03	-0.87 / 1.19

Supplementary Table 7. Crystal structure of $Li_{3.3}SnS_{3.3}Cl_{0.7}$.

Site	x	y	z	B_{iso} (Å^2)	*sof*	Wyckoff position
S	1/3	2/3	0.378(3)	2.5(4)	0.823(8)	2*b*
Cl	1/3	2/3	0.378(3)	1.2(3)	0.175(8)	2*b*
Sn	1/3	2/3	0	2.4(4)	0.242(3)	2*b*
Li1	1/3	2/3	-0.013(1)	3.3(5)	0.760(3)	2*b*
Li2	0	0	0.079(4)	3.3(5)	0.092(8)	2*b*

Supplementary Table 8. Selected interatomic distances and angles for $Li_{3.3}SnS_{3.3}Cl_{0.7}$.

Atom	Distance around atom (Å)			Angles around atom (°)				Coordination
Sn	Sn	S/Cl	2.4016(3) ×3	S/Cl	Sn	S/Cl	108.66(2) ×3	4
	Sn	S/Cl	2.4207(3)	S/Cl	Sn	S/Cl	110.27(2) ×3	
Li1	Li1	S/Cl	2.3961(3) ×3	S/Cl	Li1	S/Cl	106.83(2) ×3	4
	Li1	S/Cl	2.4823(3)	S/Cl	Li1	S/Cl	111.98(2) ×3	
Li2	Li2	S/Cl	2.9781(3) ×3	S/Cl	Li2	S/Cl	83.659(13) ×3	6
	Li2	S/Cl	2.6246(2) ×3	S/Cl	Li2	S/Cl	88.50(1) ×6	
				S/Cl	Li2	S/Cl	169.46(2) ×3	
				S/Cl	Li2	S/Cl	98.35(2) ×3	

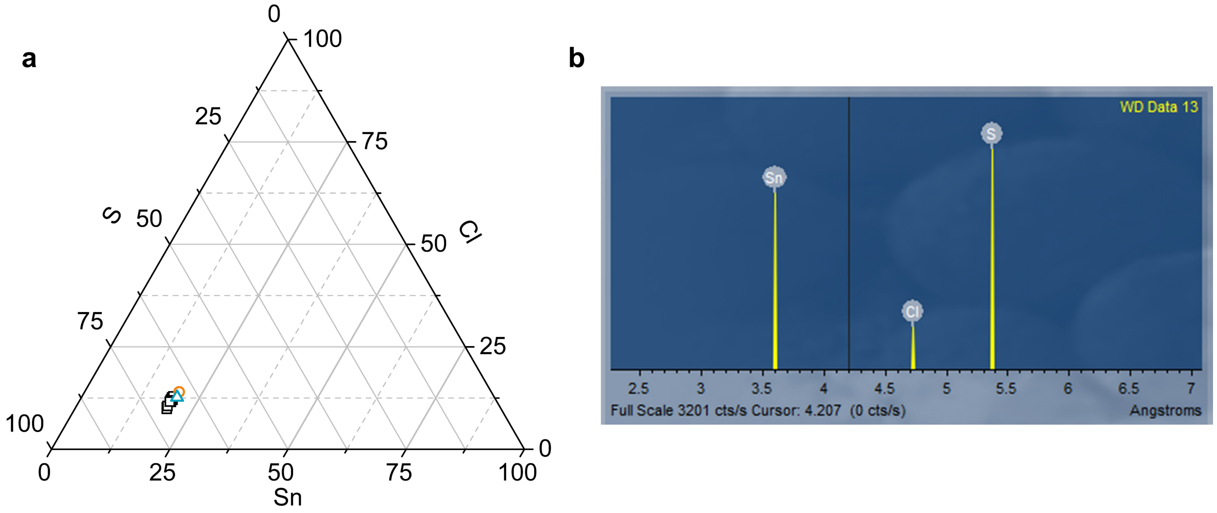

Supplementary Figure 8. Elemental analysis via SEM-WDX and ICP-AES on $Li_{3.3}SnS_{3.3}Cl_{0.7}$ **a** shows datapoints collected from 15 different areas (black squares) and the expected composition of $Li_{3.3}SnS_{3.3}Cl_{0.7}$ (orange circle) showing that the composition measured by WDX agrees well with that expected and that the composition is homogeneous throughout the material. Analysis by WDX yields an averaged measured ratio of 1.000(6):3.57(4):0.61(4) for Sn:S:Cl close to the expected values of 1:3.3:0.7. The composition of $Li_{3.305(14)}Sn_{1.000(9)}S_{3.317(44)}Cl_{0.6269(8)}$ measured by ICP-AES (blue triangle) also agrees well with the expected values. **b** shows an example WDX spectrum measured from one of the fifteen areas with peaks observed for Sn, Cl and S.

Li ion dynamics

AC-Impedance spectroscopy

The first shoulder appearing at high frequency on the Nyquist plot on Figure 4a (main text)

corresponds to the contribution of the bulk conductivity whereas the semicircle at lower frequency is characteristic of the grain boundary response to the conductivity. Despite both semicircles not being fully resolved, it is possible to fit the data using an equivalent circuit taking both contributions into account (inset Figure 4a). The estimated capacitances from the value of the admittance of the Constant Phase Element (CPE) using Hsu and Mansfeld's equation,[37] are 3.2(6) × 10^{-12} F and 1.1(2) × 10^{-11} F, for the first and second semi-circles, respectively, which are consistent with bulk and grain boundary contributions.[38] AC-impedance was measured over the temperature range 30-125 °C and the bulk conductivity was extracted at each temperature in the same way as described above for the room temperature conductivity.

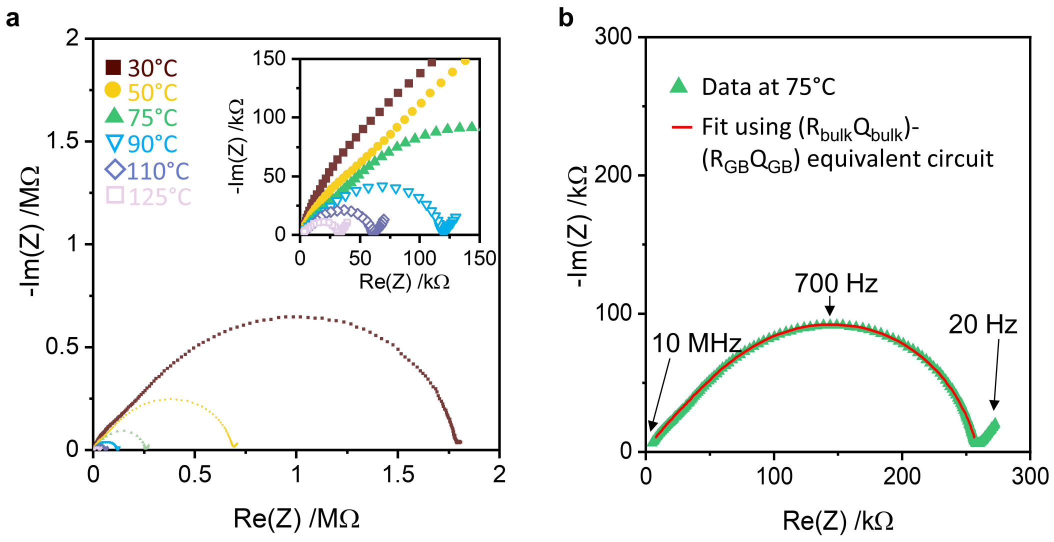

Supplementary Figure 9. AC-impedance response of $Li_{3.3}SnS_{3.3}Cl_{0.7}$ from 30 to 125 °C. a Nyquist plots of the impedance response at variable temperature of $Li_{3.3}SnS_{3.3}Cl_{0.7}$. **b** Nyquist plot at 75°C and fit using the equivalent circuit shown in Figure 4a (see Main Text).

The results of these fits are shown in Supplementary Table 9.

Supplementary Table 9. Results of the fits to the impedance data collected in the 30-125 °C temperature range on a cylindrical pellet of $Li_{3.3}SnS_{3.3}Cl_{0.7}$ with surface S = 0.50(5) cm^2 and thickness e = 0.10(1) cm.

Temp /°C	χ^2	R_{bulk} /Ω	Q_{bulk}	n_{bulk}	R_{GB} /Ω	Q_{GB}	n_{GB}
**110**	3.59 × 10^{-5}	8.7(1) × 10^3	--	--	5.28 × 10^4	5.9(1) × 10^{-11}	0.859(2)

**90**	5.01×10^{-5}	$1.40(2) \times 10^{4}$	--	--	$1.14(3) \times 10^{4}$	$6.1(2) \times 10^{-11}$	0.849(2)
**70**	1.40×10^{-5}	$3.13(6) \times 10^{4}$	--	--	$2.62(7) \times 10^{4}$	$5.27(9) \times 10^{-11}$	0.853(2)
**50**	5.76×10^{-3}	$9(1) \times 10^{4}$	$1.0(2) \times 10^{-10}$	0.76(1)	$6(2) \times 10^{5}$	$4.8(9) \times 10^{-11}$	0.85(2)
**30**	5.64×10^{-3}	$1.4(2) \times 10^{5}$	$2.8(6) \times 10^{-11}$	0.84(2)	1.80×10^{6}	$6.6(1) \times 10^{-11}$	0.81(2)

Determination of the electronic conductivity through DC polarization measurements

Determination of the electronic conductivity contribution was performed through DC polarization measurements, which consist of applying a steady voltage while measuring the current response as a function of time. The value of the current at $t = 0$ accounts for both ionic and electronic conductivities and then it decreases exponentially as the ions polarize at the ion blocking electrode (Supplementary Fig. 10a-e). At $t = \infty$, all ions are polarized, and only electrons contribute to the conductivity. Changing the value of the steady voltage and recording the value of the current after long enough equilibrium time enables construction of the I-V curve. The value of the electronic conductivity is then extracted through Ohm's law (Supplementary Fig. 10f). At room temperature, the electronic conductivity is $\sigma_e = 1.1(1) \times 10^{-8}$ S cm^{-1}. The electronic conductivity therefore contributes to around 1% of the total conductivity ($\sigma_{tot} = 12.4(1) \times 10^{-6}$ S cm^{-1}) and can be considered negligible.

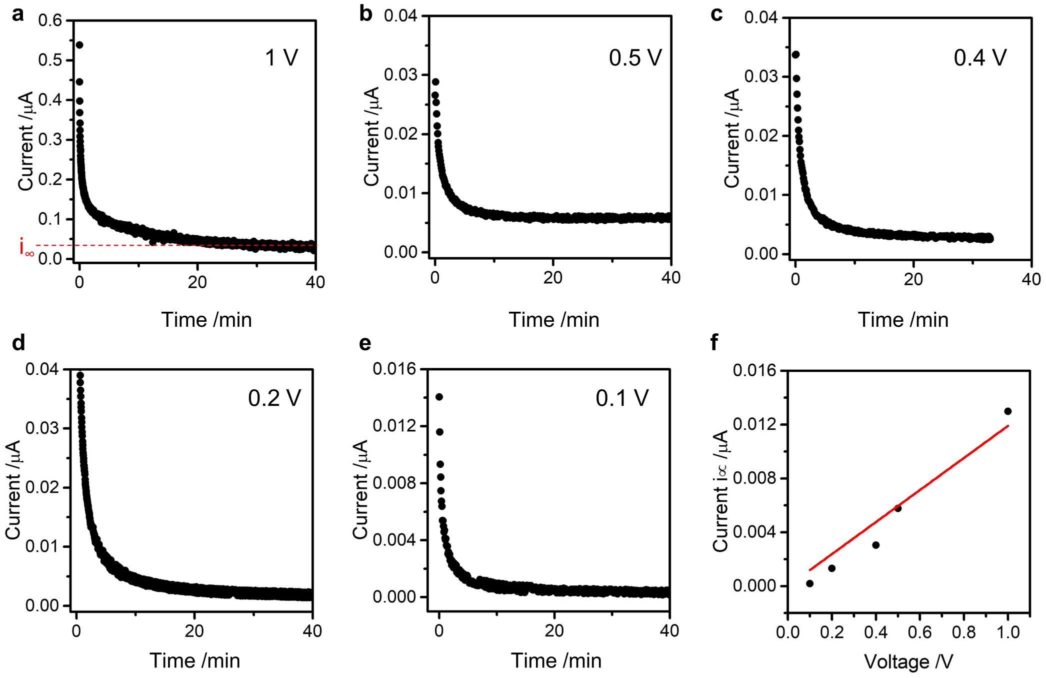

Supplementary Figure 10. a-e I-t curve measured at different steady applied voltages (value in legend) and **f** I-V curve of the electronic contribution. A fit using Ohm's law yields a total electronic conductivity 1.1(1) × 10^{-8} S cm^{-1}.

Prior to the DC polarization, EIS measurements were performed at room temperature using a Biologic VSP-300 potentiostat/galvanostat, which enables the collection of the impedance spectra down to a low frequency of 1 mHz, permitting visualization the Warburg tail at low frequency region (Supplementary Fig. 11). Fitting of the data points using a (R-CPE) equivalent electrical circuit yields a total conductivity of 1.0(4) × 10^{-6} S cm^{-1} close to the value obtained using the

Keysight instrument (data collected to 20 Hz, cf. Figure 4, main text).

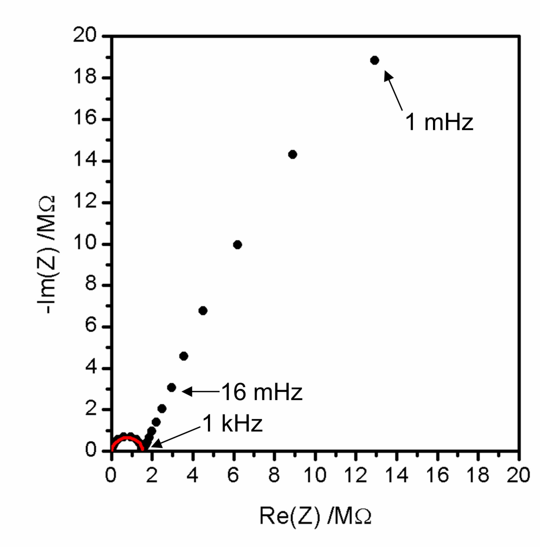

Supplementary Figure 11. EIS of the Au|$Li_{3.3}SnS_{3.3}Cl_{0.7}$|Au ion-blocking electrode measured at room temperature from 3 MHz to 1 mHz. The red line corresponds to the fit of the data points using a (R-CPE) parallel equivalent electrical circuit yielding R = 1.55(2) MΩ, Q = 3.87(7) and n = 0.91(1).

Evidence of ionic conductivity and stability against Li metal through Li plating/stripping in a Li|$Li_{3.3}SnS_{3.3}Cl_{0.7}$|Li symmetric cell

The voltage profile of the Li|$Li_{3.3}SnS_{3.3}Cl_{0.7}$|Li symmetric cell upon repeated Li plating/stripping at room temperature, 323 K and 343 K over 400 h, is shown in Figure 4e, and Supplementary Fig. 12a. The transport of Li^+ through the $Li_{3.3}SnS_{3.3}Cl_{0.7}$ solid-state electrolyte, as well as across the $Li_{3.3}SnS_{3.3}Cl_{0.7}$|$Li^0$ interface, can be confirmed. An initial polarization of ±1.28 V was observed at a low current density of 10 μA cm^{-2} at room temperature due to the moderate ionic conductivity of the material. At elevated temperatures, the polarization is largely reduced, confirming improved ionic conductivity. The change in overpotential can be used to evaluate the Li|$Li_{3.3}SnS_{3.3}Cl_{0.7}$ interfacial reactions and possible degradation of $Li_{3.3}SnS_{3.3}Cl_{0.7}$. During the initial 50 h, the overpotential rises gently, in line with the increased diameter of the semicircle over cycling observed from the EIS (Supplementary Fig. 12), indicating the formation of an interphase layer. Interestingly, the

overpotentials remain constant over the subsequent plating/stripping, suggesting a stable interphase layer is formed. This promising result is in contrast with what has been observed in the Sb- and As-doped Li_4SnS_4 material,[39,40] which showed irregular voltage profiles over 20 h. After galvanostatic stripping and plating, the symmetric cell was disassembled. At the Li|$Li_{3.3}SnS_{3.3}Cl_{0.7}$ interface, the colour of $Li_{3.3}SnS_{3.3}Cl_{0.7}$ pellet became darker. XRD measurements of $Li_{3.3}SnS_{3.3}Cl_{0.7}$ material extracted from the bulk pellet and also from this interfacial region show negligible change when compared against the as-synthesised powder (Supplementary Fig. 13). No difference can be seen from the Raman spectra between the cycled bulk pellet and the pristine $Li_{3.3}SnS_{3.3}Cl_{0.7}$ (Supplementary Fig. 14). However, the dark colour powders scraped from the Li surface show new Raman bands, which can be tentatively assigned to S-S-S bending (*i.e.*, S_8, 158/215 cm^{-1}),[41] Sn-S (185 cm^{-1}),[42] and SCl_2/S_3^- (~525 cm^{-1})[43] that have been observed at the interface between Li^0 and sulfide-based solid state electrolytes.[41] The mixed S-Cl anion material enhances stability properties against Li metal compared to the monoanionic sulfide Li_4SnS_4 without degrading the ionic conductivity.

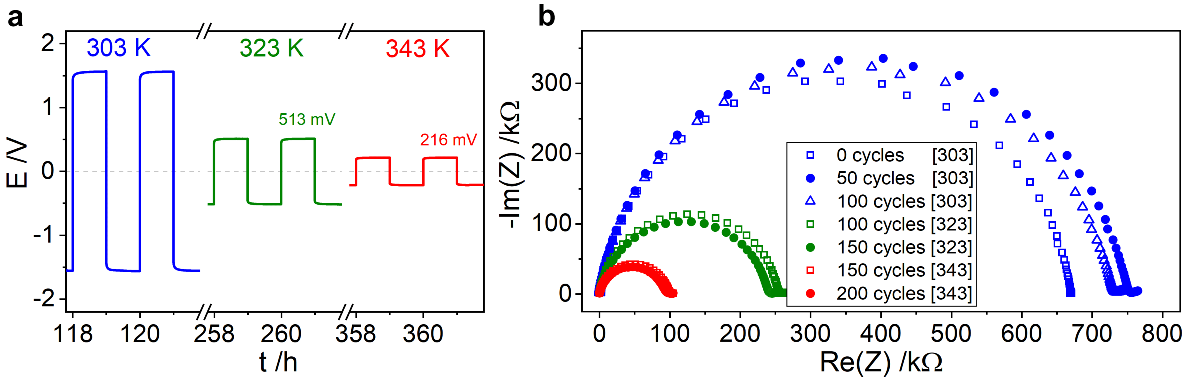

Supplementary Figure 12. Galvanostatic Li plating/stripping of a symmetric Li|$Li_{3.3}SnS_{3.3}Cl_{0.7}$|Li cell. **a** Enlarged snapshots of voltage profiles at the three studied temperatures. **b** Electrochemical impedance spectra collected at 50 cycle intervals for the cell shown in Figure 4e

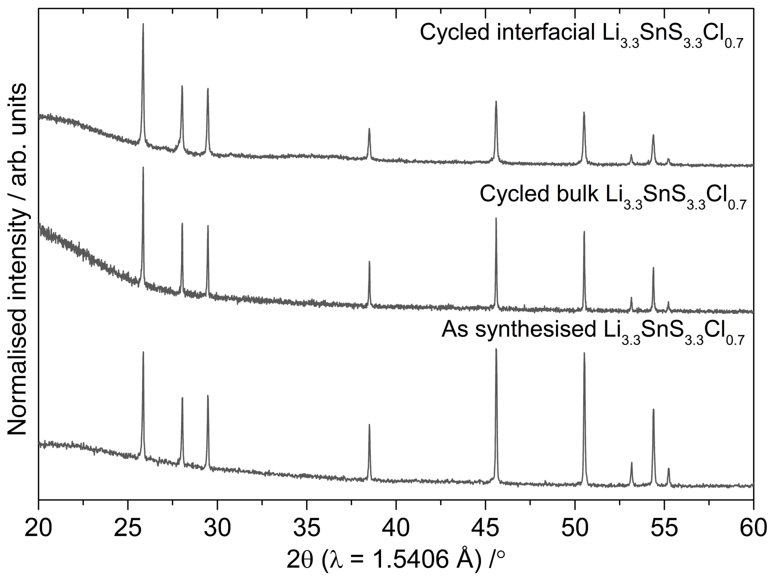

Supplementary Figure 13. *Ex situ* investigation of $Li_{3.3}SnS_{3.3}Cl_{0.7}$ after galvanostatic Li plating/stripping. Laboratory XRD patterns show comparison between as-synthesized $Li_{3.3}SnS_{3.3}Cl_{0.7}$, material extracted from the bulk of the cycled pellet, and powder which was scraped from the interfacial region of the Li|$Li_{3.3}SnS_{3.3}Cl_{0.7}$|Li cell. This interfacial powder was mixed with amorphous boron (B) and sealed in a 0.5 mm diameter borosilicate capillary for measurement.

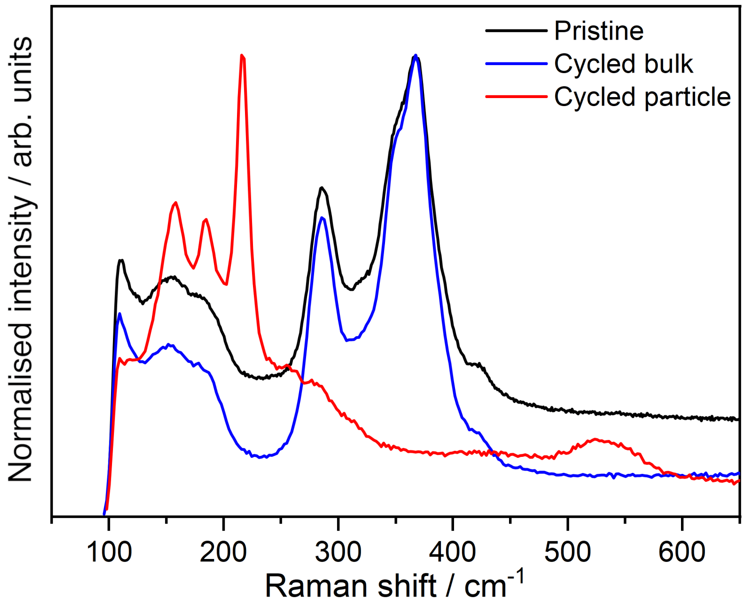

Supplementary Figure 14. Ex situ Raman spectra of $Li_{3.3}SnS_{3.3}Cl_{0.7}$ after galvanostatic Li plating/stripping. Raman microscopy of the as-synthesized pristine $Li_{3.3}SnS_{3.3}Cl_{0.7}$ (black), the bulk solid electrolyte after cycling (blue), and of the small dark particles within the bulk cycled powder visible under the microscope (red). No peaks were observed in the higher wavenumber regions 700-4000 cm^{-1} for any of the studied materials.

Nuclear Magnetic Resonance Spectroscopy

In static ^7Li solid-state NMR spectra the absence of mobility is displayed through a broadening of the 1/2 ↔ −1/2 central transition arising from the strong homonuclear ^7Li -^7Li dipolar coupling interactions. At low temperatures the material is said to be in the rigid lattice regime, in this regime spectra dominated by dipolar broadening are observed. This effect is present at approximately 165 K, where the line width of the central transition is approximately 5.2 kHz for $Li_{3.3}SnS_{3.3}Cl_{0.7}$. As the temperature is increased the dipolar interactions are continuously averaged due to the increasing motion of the Li spins causing the spectra to narrow significantly (Supplementary Fig. 9). This effect can be seen by plotting the full width half maximum of the peak in function of the temperature (Figure 4b), where the onset of motional narrowing, T_{onset} occurs at around 200 K. Using an expression introduced by Waugh and Fedin[44] relating the onset temperature of motional narrowing with the activation energy of the diffusion process, given by:

$$E_a = 1.67 \times 10^{-3}\, T_{onset} \qquad \text{(Supplementary Equation 4)}$$

an activation energy of approximately 0.3 eV can be estimated for $Li_{3.3}SnS_{3.3}Cl_{0.7}$. The peak width decreases significantly as the temperature is increased above ∼200 K, down to a minimum line width of approximately 750 Hz where the homonuclear dipolar interactions are completely averaged out.

As $Li_{3.3}SnS_{3.3}Cl_{0.7}$ appears to be in the fast-motional regime at room temperature, this is a reasonable explanation for the absence of two distinct resonances attributed to the material in the ^6Li MAS NMR spectrum. At room temperature rapid exchange between tetrahedral and octahedral Li sites will occur on the NMR timescale leading to the observation of a single resonance.

The temperature dependence of the ^7Li SLR in the laboratory (T_1^{-1}) and rotating frame ($T_{1\rho}^{-1}$)

under static conditions were monitored using the saturation recovery and spin lock pulse sequences. These measurements were performed in order to obtain values for the activation energy, conductivity and dimensionality of the Li diffusion. The SLR $T_{1\rho}^{-1}$ rates are purely induced by diffusion processes initially increasing with temperatures above room temperature before decreasing thereby passing through a maximum at temperatures that are characteristic of the Li correlation rates τ_0^{-1} (that is the average Li jump rates τ^{-1}) being equal to the spin-lock frequencies ω_1, *i.e.* $2\omega_I \approx \tau_c^{-1} = \tau^{-1}$. Accessing these maxima at different ω_I enables different NMR-derived jump rates τ^{-1} to be obtained at different temperatures (Figure 4c). τ^{-1} from NMR line narrowing experiments and relaxometry experiments are plotted against reciprocal temperature in Supplementary Fig. 9. Fitting to $\tau^{-1} = \tau_0^{-1}exp(-E_a/(RT))$ yields an activation barrier of 0.23(6) eV for $Li_{3.3}SnS_{3.3}Cl_{0.7}$.

The dimensionality of the Li^+ ion diffusion can be accessed from the frequency-dependence of the high temperature limits of the SLR $T_{1\rho}^{-1}$ values and follows characteristic relationships with one, two, and three-dimensional diffusion in solids being proportional to $(\tau/\omega)^{0.5}$, $\tau ln(1/\omega\tau)$, or τ, respectively (where τ and ω are the correlation times and probe frequencies, respectively) for which the corresponding fit is shown at various temperatures in Supplementary Fig. 17. NMR conductivity σ_{NMR} can be estimated from the Li jump rates τ^{-1} using the combined Nernst-Einstein and Einstein-Smoluchowski equations:

$$\sigma_{NMR} = \frac{\mathrm{f}}{\mathrm{H_R}}\frac{N_{\mathrm{CC}}q^2a^2}{N_{\mathrm{NN}}\mathrm{k_B T}}\frac{1}{\tau} \qquad \text{(Supplementary Equation 5)}$$

where f/H_R is the correlation factor and Haven ratio (1 for uncorrelated motion), N_{CC}/N_{NN} is the number of charge carriers per unit cell volume (0.092 Å^{-3}), q is the ionic charge of Li, a is the closest Li-Li jump distance at room temperature (2.4 Å), extracted from the crystal structure determined via diffraction experiments.

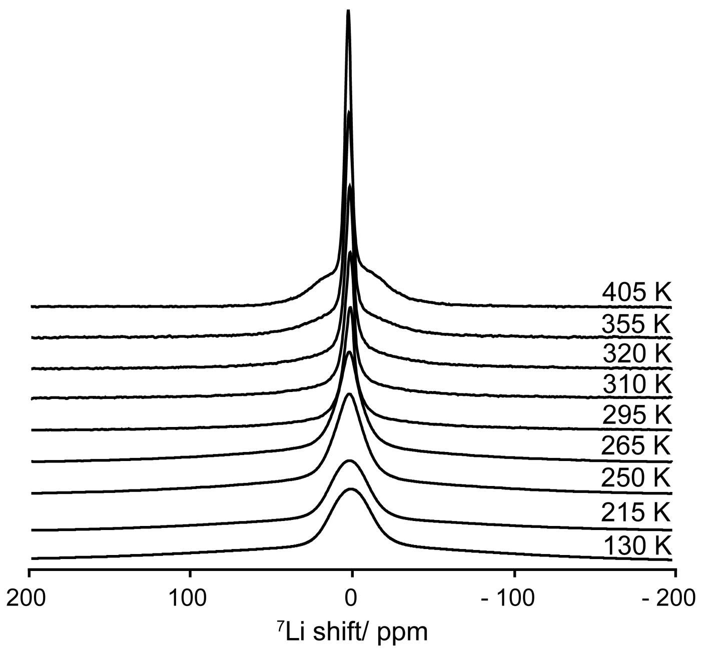

Supplementary Figure 15. ^{7}Li NMR spectra as a function of temperature for $Li_{3.3}SnS_{3.3}Cl_{0.7}$.

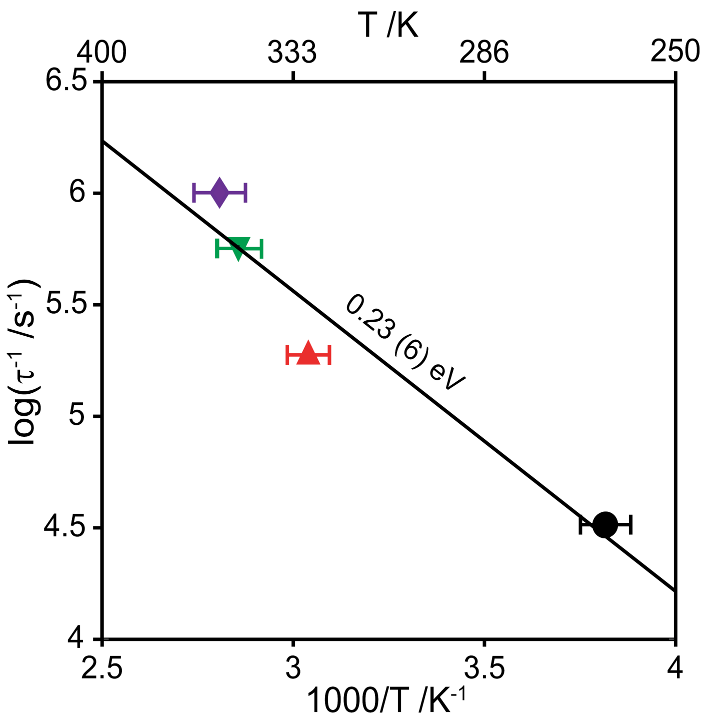

Supplementary Figure 16. Arrhenius plot of Li jump rates τ^{-1}. Data were extracted from the onset of ^{7}Li line narrowing of the variable temperature ^{7}Li NMR spectra (black circle, Figure 4b) and SLR rates in the rotating frame ($T_{1\rho}^{-1}$) experiments (Figure 4c) at spin lock frequencies $\omega_1/2\pi$ of 15 (red triangle), 45 (green inverted triangle) and 80 kHz (purple diamond), respectively. The black error bar associated with the temperature is calculated from the broadening of the isotropic peak of the chemical shift thermometer $Pb(NO_3)_2$ as explained above. Errors in the jump rate τ^{-1} are within the

data points.

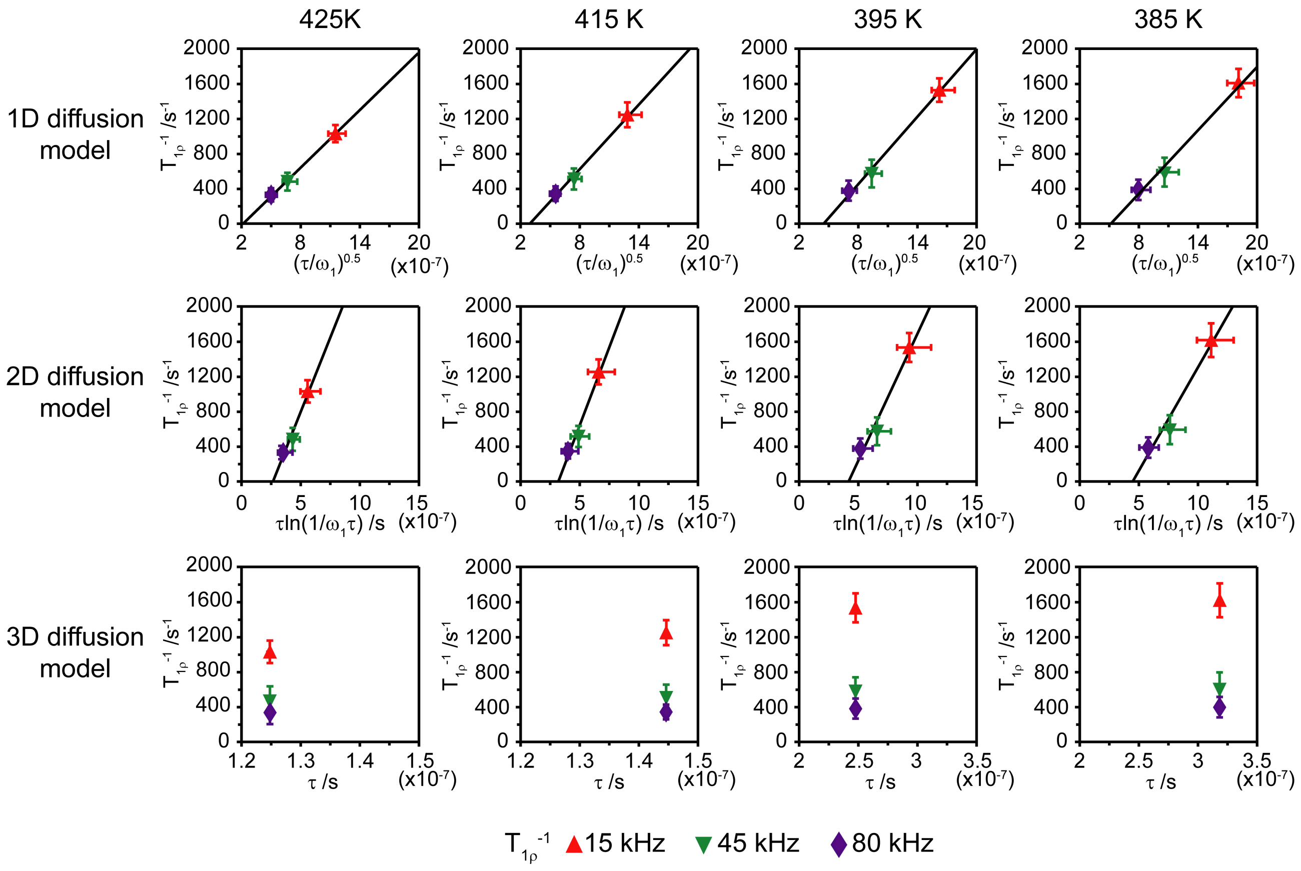

Supplementary Figure 17. Frequency dependence of the NMR SLR $T_{1\rho}^{-1}$ rates at 425, 415, 395 and 385 K for one, two and three-dimensional models, at spin lock frequencies $\omega_l/2\pi$ of 15 (red triangle), 45 (green inverted triangle), 80 kHz (purple diamond), and average Li+ jump times τ from $T_{1\rho}^{-1}$ maxima (Figure 4c). The solid lines correspond to linear fits of $(\tau/\omega)^{0.5}$ and $\tau ln(1/\omega\tau)$ for one and two-dimensional diffusion, respectively. The frequency dependence of the SLR $T_{1\rho}^{-1}$ rates (also shown in Figure 4c) clearly rules out the possibility of three-dimensional diffusion. Errors in the spin-lock frequencies ω_l are estimated to be 10% while the errors in the correlation time τ are extracted from the fit in Supplementary Fig. 16. Errors in $T_{1\rho}^{-1}$ are obtained from the outputs of the fits to Supplementary Equation 3.

Supplementary Table 10. Previously reported Li pathways in hcp sulphides described in terms of interstitial site occupancy and dimensionality.

Composition	Interstitial site involvement in the hopping pathway[45]	Dimensionality	References
γ-Li_3PS_4	T – T (in the (*ac*) plane)	2D	Pan *et al.*[46]
Li_4GeS_4	O – T – O (along *b*)	1D	Al-Qawasmeh *et al.*[47]<br>Minafra *et al.*[48]
Li_4SnS_4	T – O – T (along *b*)	3D	Al-Qawasmeh *et al.*[47]

	T – O – T (along *a*)<br>T – T (along *c*)		Minafra *et al.*[48]
$Li_{4.4}Al_{0.4}Ge_{0.6}S_4$	T – O – T (in the (*ab*) plane)	2D	Leube *et al.*[49]

Exploration of Li-Mg-S-Cl quaternary phase fields

Computational and Experimental Results

Li-Mg-S-Cl is ranked #4 in the list of unexplored phase fields by the variational autoencoder (Supplementary Table 3). Compositions in this phase field were sampled computationally prior to synthesis using a method similar to that described in the main text, identifying a low energy region which lead to the discovery of new compounds (Supplementary Fig. 18). The variational autoencoder identified and ranked highly this series of compounds that had not been discovered in the previous four decades since the initial report of ionic conductivity in Li_2MgCl_4.[50,51]

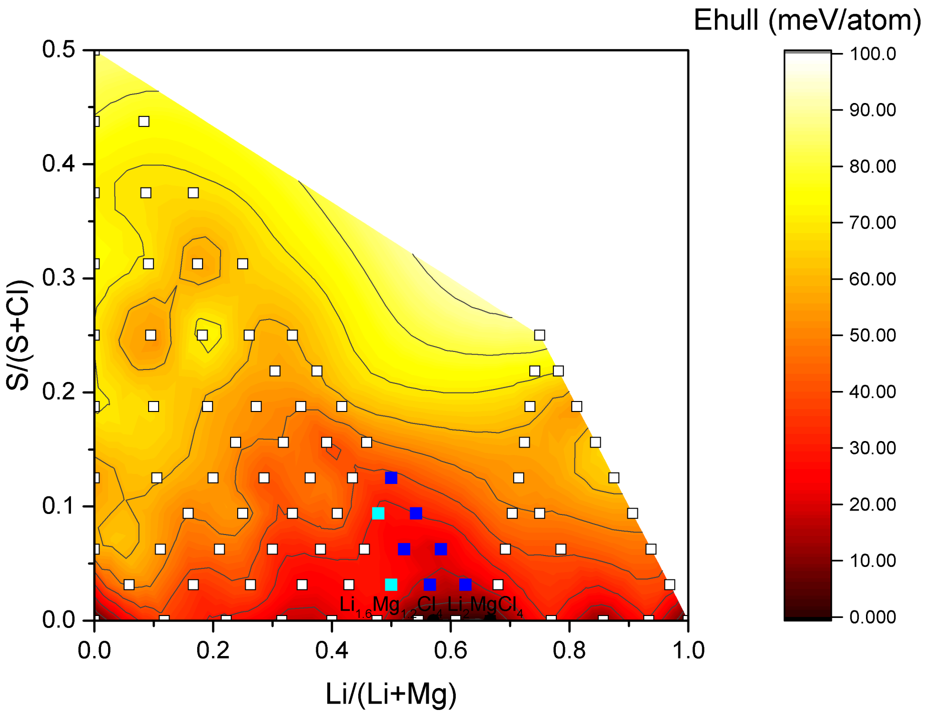

Supplementary Figure 18. Computational and experimental exploration of Li-Mg-S-Cl phase field. Contour plot of energy above the convex hull for the computationally explored set of compositions (white squares) in the Li-Mg-S-Cl phase field. The compositions marked by blue squares were successfully synthesized as single-phase powders through a conventional solid-state route, whilst the compositions shown by cyan squares were single-phase only after quenching.

Experimental syntheses were carried out by grinding LiCl (Merck, 99.99%), Li_2S (Merck, 99.98%) and $MgCl_2$ (Merck, 99.9%) together with a pestle and mortar before pressing into pellets and sealing in carbon-coated evacuated (<10^{-4} mbar) sealed silica ampoules and heating to 600 °C for 12 hrs using heating and cooling rates of 5°C min^{-1}. Powder XRD patterns measured from samples of these low computed energy compositions indicate successful formation of new single-phase materials which can be accessed through $Li_{2-2x}Mg_{1+x+y}Cl_{4-y}S_y$, where x = 0, 0.125 and 0.25, and y ≤ 0.5. These compositions maintain a total anion content of 4, but introduce cation vacancies up to a maximum content of 0.25 (8.3% of the total number of cations). All of the observed reflections in powder patterns from the new $Li_{2-2x}Mg_{1+x+y}Cl_{4-y}S_y$ series can be indexed to a cubic unit cell with space group symmetry $Fd\bar{3}m$ (Supplementary Fig. 19).

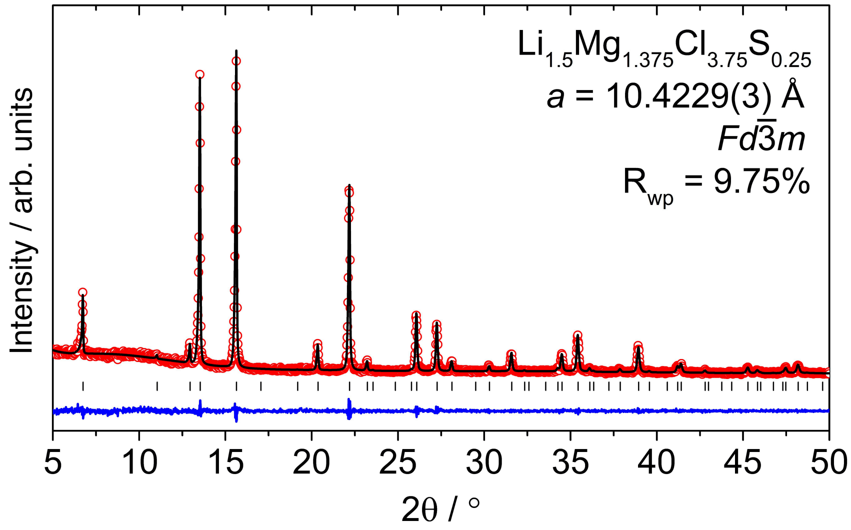

Supplementary Figure 19. Pawley fit of $Li_{1.5}Mg_{1.375}Cl_{3.75}S_{0.25}$. Pawley fit of XRD data measured from $Li_{1.5}Mg_{1.375}Cl_{3.75}S_{0.25}$ with I_{obs} (red dots), I_{calc} (black line), I_{obs} - I_{calc} (blue line), and Bragg reflections (black tick marks).

A solubility limit exists within the new $Li_{2-2x}Mg_{1+x+y}Cl_{4-y}S_y$ series. Six compositions were synthesized using the conditions described above, shown by blue squares in Supplementary Fig.

20a. Synthesis attempts at other compositions (green squares and triangles in Supplementary Fig. 20a) were unsuccessful, resulting in significant amounts of MgS or $MgCl_2$ as secondary phases and no further change in lattice parameter of the majority cubic phase. Quenching of powder samples by placing sealed tubes into a water bath from synthesis temperature, rather than cooling at a controlled rate, was used to promote further solubility of Mg through entropic mechanisms. This enabled the isolation of two further compositions (cyan squares in Supplementary Fig. 20a) increasing the solubility of Mg to $Li_{1.375}Mg_{1.5}Cl_{3.625}S_{0.375}$ or increasing the cation vacancy content x = 0.25 in the compound $Li_{1.375}Mg_{1.375}Cl_{3.875}S_{0.125}$. Incorporation of 1.5Mg and 0.25 vacancies per formula unit of Li_2MgCl_4 is a significant increase compared to a previous study which indicated the solubility limit was $Li_{1.6}Mg_{1.2}Cl_4$ with 0.2 cation vacancies,[29] and directly results from anion mixing via incorporation for the second anion (S^{2-}) originally identified and ranked by the variational autoencoder.

The phase pure $Li_{2-2x}Mg_{1+x+y}Cl_{4-y}S_y$ materials (x = 0, 0.125 and 0.25 and y ≤ 0.5; Supplementary Table 11) obtained through these synthesis methods all obey Vegard's law where extracted lattice parameters follow a linear trend as a function of doping. Replacement of Cl^- for S^{2-}, Li^+ for Mg^{2+} and introduction of cation vacancies all play a role in these behaviours (Supplementary Fig. 20b and c). Monotonic decreases in lattice parameter are observed with increasing y (S content) for samples with the same number of cation vacancies; the effect of substituting Li^+ with smaller Mg^{2+} ($r_{Li}{}^+$ = 0.76 Å, $r_{Mg}{}^{2+}$ = 0.72 Å) is more significant that the lattice expansion expected from replacing Cl^- with S^{2-} ($R_{Cl}{}^-$ = 1.81 Å, $r_S{}^{2-}$ = 1.84 Å)[52]. A monotonic decrease in lattice parameter is observed with increasing x (cation vacancy content) at fixed y (S content) also, highlighting the impact of cation

vacancies on unit cell size in these materials with a cubic close-packed anion lattice.

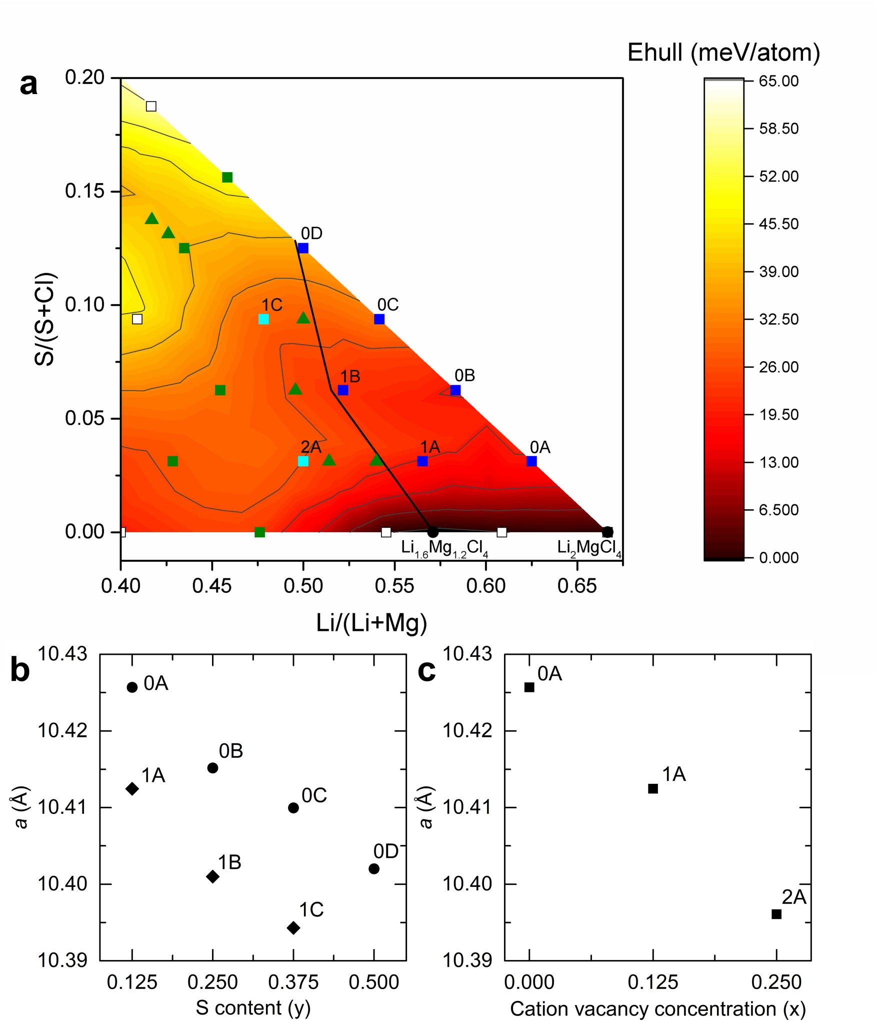

Supplementary Figure 20. Solubility limit of Mg in $Li_{2-2x}Mg_{1+x+y}Cl_{4-y}S_y$ and lattice parameters. a Low energy region of Li-Mg-S-Cl computed phase field showing attempted and successful synthesis. Compositions shown as blue squares were synthesized as single-phase powders through a conventional solid-state route, whilst the compositions shown as cyan squares were single-phase only after quenching. Green squares and triangles represent unsuccessful synthesis attempts. Black line represents proposed solubility limit of Mg and cation vacancies in $Li_{2-2x}Mg_{1+x+y}Cl_{4-y}S_y$ accessible through conventional solid-state routes. Labels infer x and y in $Li_{2-2x}Mg_{1+x+y}Cl_{4-y}S_y$, where 0, 1 and 2 correspond to x values (vacancy content) of 0, 0.125 and 0.25, respectively, while A, B, C, D correspond to y values (S content) of 0.125, 0.25, 0.375 and 0.5. **b** lattice parameters plotted as a function of y (S content) for compositions with no cation vacancies (circles) and 0.125 cation vacancies (diamonds). **c** lattice parameters plotted as a function of x (cation vacancy content) for compositions with y = 0.125.

Compositions measured through ICP-AES by Mikroanalytisches Labor Pascher (*cf.* Methods) for each material in the $Li_{2-2x}Mg_{1+x+y}Cl_{4-y}S_y$ series show reasonable agreement with the nominal compositions (Supplementary Table 11); *e.g.*, an overall composition of $Li_{1.506(1)}Mg_{1.392(4)}Cl_{3.763(8)}S_{0.237(2)}$ is obtained for $Li_{1.5}Mg_{1.375}Cl_{3.75}S_{0.25}$. Though the sulphur content

for each material is slightly underestimated, likely due to the loss of some sulphur through evolutions of H_2S upon dissolution, the results provide clear evidence for combination of multiple anions (Cl and S) in these materials.

Supplementary Table 11. Nominal and measured compositions for phase-pure materials studied in the $Li_{2-2x}Mg_{1+x+y}Cl_{4-y}S_y$ series obtained through ICP-AES. Values are normalised to a total anion content of 4.

Nominal composition	Code (Fig. S18)	Li	Mg	Cl	S	Synthesis
Li_2MgCl_4	-	2.141(20)	0.945(4)	4.000(8)	-	Solid-state
No cation vacancies; x = 0						
$Li_{1.875}Mg_{1.125}Cl_{3.875}S_{0.125}$	0A	1.989(5)	1.139(4)	3.899(31)	0.101(6)	Solid-state
$Li_{1.75}Mg_{1.25}Cl_{3.75}S_{0.25}$	0B	1.697(8)	1.279(4)	3.739(10)	0.207(2)	Solid-state
$Li_{1.625}Mg_{1.375}Cl_{3.625}S_{0.375}$	0C	1.568(1)	1.399(4)	3.689(10)	0.311(3)	Solid-state
$Li_{1.5}Mg_{1.5}Cl_{3.5}S_{0.5}$	0D	1.471(5)	1.523(4)	3.568(34)	0.432(2)	Solid-state
Cation vacancies; x = 0.125						
$Li_{1.625}Mg_{1.25}Cl_{3.875}S_{0.125}$	1A	1.705(2)	1.218(4)	3.909(39)	0.091(3)	Solid-state
$Li_{1.5}Mg_{1.375}Cl_{3.75}S_{0.25}$	1B	1.506(1)	1.392(4)	3.673(8)	0.273(2)	Solid-state
$Li_{1.375}Mg_{1.5}Cl_{3.625}S_{0.375}$	1C	1.385(7)	1.532(4)	3.700(5)	0.300(2)	Quenching
Cation vacancies; x = 0.25						
$Li_{1.375}Mg_{1.375}Cl_{3.875}S_{0.125}$	2A	1.395(15)	1.402(4)	3.959(5)	0.041(2)	Quenching

Clear evidence for new quaternary materials in the Li-Mg-S-Cl phase field, ranked highly by the VAE (ranked #4, Supplementary Table 3), is provided by the results of synthetic exploration presented here. The series $Li_{2-2x}Mg_{1+x+y}Cl_{4-y}S_y$ (where x = 0, 0.125 and 0.25 and y ≤ 0.5) is discovered through anion mixing and incorporation of cation vacancies.

Supplementary References

1. Chalapathy, R. & Chawla, S. Deep Learning for Anomaly Detection: A Survey. *arXiv:1901.03407 [cs, stat]* (2019).
2. Marques, H. O., Campello, R. J. G. B., Sander, J. & Zimek, A. Internal Evaluation of Unsupervised Outlier Detection. *ACM Trans. Knowl. Discov. Data* **14**, 47:1-47:42 (2020).
3. Zhao, Y., Nasrullah, Z. & Li, Z. PyOD: A Python Toolbox for Scalable Outlier Detection. *J. Mach. Learn. Res.* **20**, 1–7 (2019).
4. Srivastava, N., Hinton, G., Krizhevsky, A., Sutskever, I. & Salakhutdinov, R. Dropout: A Simple Way to Prevent Neural Networks from Overfitting. *J. Mach. Learn. Res.* **15**, 1929–1958 (2014).
5. Perez-Cruz, F. Kullback-Leibler Divergence Estimation of Continuous Distributions. in *2008 IEEE International Symposium on Information Theory* 1666–1670 (2008). doi:10.1109/ISIT.2008.4595271.
6. Kingma, D. P. & Ba, J. Adam: A Method for Stochastic Optimization. *arXiv:1412.6980 [cs]* (2017).
7. Zhou, C. & Paffenroth, R. C. Anomaly Detection with Robust Deep Autoencoders. in *Proceedings of the 23rd ACM SIGKDD International Conference on Knowledge Discovery and Data Mining* 665–674 (Association for Computing Machinery, 2017). doi:10.1145/3097983.3098052.
8. Palacio-Niño, J.-O. & Berzal, F. Evaluation Metrics for Unsupervised Learning Algorithms. *arXiv:1905.05667 [cs, stat]* (2019).
9. Wang, X., Peng, D., Hu, P. & Sang, Y. Adversarial Correlated Autoencoder for Unsupervised Multi-View Representation Learning. *Knowl. Based Syst.* **168**, 109–120 (2019).

10. Gong, D. *et al.* Memorizing Normality to Detect Anomaly: Memory-Augmented Deep Autoencoder for Unsupervised Anomaly Detection. in *2019 IEEE/CVF International Conference on Computer Vision (ICCV)* 1705–1714 (2019). doi:10.1109/ICCV.2019.00179.

11. Izenman, A. J. Recent Developments in Nonparametric Density Estimation. *J. Am. Stat. Assoc.* **86**, 205–224 (1991).

12. Jha, D. *et al.* ElemNet : Deep Learning the Chemistry of Materials From Only Elemental Composition. *Sci. Rep.* **8**, 1–13 (2018).

13. Ward, L., Agrawal, A., Choudhary, A. & Wolverton, C. A General-Purpose Machine Learning Framework for Predicting Properties of Inorganic Materials. *Npj Comput. Mater.* **2**, 16028 (2016).

14. Glawe, H., Sanna, A., Gross, E. K. U. & Marques, M. A. L. The Optimal One Dimensional Periodic Table: a Modified Pettifor Chemical Scale from Data Mining. *New J. Phys.* **18**, 093011 (2016).

15. Nagle, J. K. Atomic Polarizability and Electronegativity. *J. Am. Chem. Soc.* **112**, 4741–4747 (1990).

16. FullProf Suite - Crystallographic Tool for Rietveld, Profile Matching & Integrated Intensity Refinements of X-Ray and/or Neutron Data, http://www.ill.eu/sites/fullprof/ (2006).

17. Rodríguez-Carvajal, J. *Study of Micro-Structural Effects by Powder Diffraction Using the Program FULLPROF, http://www.cdifx.univ-rennes1.fr/fps/Microstructural_effects.pdf.*

18. Momma, K. & Izumi, F. *Evaluation of Algorithms and Weighting Methods for MEM Analysis from Powder Diffraction Data. European Powder Diffraction Conference; August 2010, Darmstadt, Germany* 195–200 (De Gruyter, 2011). doi:10.1524/9783486991321-034.

19. Smaalen, S. van, Palatinus, L. & Schneider, M. The Maximum-Entropy Method in Superspace. *Acta Crystallogr. Sect. A: Found. Crystallogr.***59**, 459–469 (2003).

20. Weber, D. A. *et al.* Structural Insights and 3D Diffusion Pathways within the Lithium Superionic Conductor $Li_{10}GeP_2S_{12}$. *Chem. Mater.* **28**, 5905–5915 (2016).

21. Nishimura, S. *et al.* Experimental Visualization of Lithium Diffusion in Li_xFePO_4. *Nat. Mater.* **7**, 707–711 (2008).

22. Yashima, M. *et al.* Conduction path and Disorder in the Fast Oxide-Ion Conductor $(La_{0.8}Sr_{0.2})(Ga_{0.8}Mg_{0.15}Co_{0.05})O_{2.8}$. *Chem. Phys. Lett.* **380**, 391–396 (2003).

23. Bielecki, A. & Burum, D. P. Temperature Dependence of ^{207}Pb MAS Spectra of Solid Lead Nitrate. An Accurate, Sensitive Thermometer for Variable-Temperature MAS. *J. Magn. Reson., Series A* **116**, 215–220 (1995).

24. Beckmann, P. A. & Dybowski, C. A Thermometer for Nonspinning Solid-State NMR Spectroscopy. *J. Magn. Reson.* **146**, 379–380 (2000).

25. Becker, K. D. Temperature Dependence of NMR Chemical Shifts in Cuprous Halides. *J. Chem. Phys.* **68**, 3785–3793 (1978).

26. Wu, J., Kim, N. & Stebbins, J. F. Temperature Calibration for High-Temperature MAS NMR to 913 K: ^{63}Cu MAS NMR of CuBr and CuI, and ^{23}Na MAS NMR of $NaNbO_3$. *Solid State Nucl. Magn. Reson.* **40**, 45–50 (2011).

27. Ahn, B. T. & Huggins, R. A. Synthesis and Lithium Conductivities of Li_2SiS_3 and Li_4SiS_4. *Mater. Res. Bull.* **24**, 889–897 (1989).

28. Lutz, H. D. Schnelle Lithium-Ionenleiter - Chemie und Anwendung. *Nachrichten aus Chemie, Technik und Laboratorium* **43**, 418–421 (1995).

29. Kanno, R., Takeda, Y., Takada, K. & Yamamoto, O. Ionic Conductivity and Phase Transition of the Spinel System $Li_{2-2x}M_{1+x}Cl_4$ (M = Mg, Mn, Cd). *J. Electrochem. Soc.* **131**, 469 (1984).

30. Gamon, J. *et al.* Computationally Guided Discovery of the Sulfide Li_3AlS_3 in the Li–Al–S Phase Field: Structure and Lithium Conductivity. *Chem. Mater.* **31**, 9699–9714 (2019).

31. Lim, H., Kim, S.-C., Kim, J., Kim, Y.-I. & Kim, S.-J. Structure of Li_5AlS_4 and comparison with other lithium-containing metal sulfides. *J. Solid State Chem.* **257**, 19–25 (2018).

32. Kaib, T. *et al.* New Lithium Chalcogenidotetrelates, LiChT: Synthesis and Characterization of the Li^+-Conducting Tetralithium ortho-Sulfidostannate Li_4SnS_4. *Chem. Mater.* **24**, 2211–2219 (2012).

33. Kanazawa, K. *et al.* Mechanochemical Synthesis and Characterization of Metastable Hexagonal Li_4SnS_4 Solid Electrolyte. *Inorg. Chem.* **57**, 9925–9930 (2018).

34. Holzmann, T. *et al.* $Li_{0.6}[Li_{0.2}Sn_{0.8}S_2]$ – A Layered Lithium Superionic Conductor. *Energy Environ. Sci.* **9**, 2578–2585 (2016).

35. Brant, J. A. *et al.* Fast Lithium Ion Conduction in Li_2SnS_3: Synthesis, Physicochemical Characterization, and Electronic Structure. *Chem. Mater.* **27**, 189–196 (2015).

36. Looijenga-Vos, A. & Buerger, M. J. Space-group Determination and Diffraction Symbols - Chap. 3.1. in *International Tables for Crystallography* vol. A 44–54 (2006).

37. Hsu, C. H. & Mansfeld, F. Technical Note: Concerning the Conversion of the Constant Phase Element Parameter Y0 into a Capacitance. *Corrosion* **57**, (2001).

38. Irvine, J. T. S., Sinclair, D. C. & West, A. R. Electroceramics: Characterization by Impedance Spectroscopy. *Adv. Mater.* **2**, 132–138 (1990).

39. Sahu, G. *et al.* Air-Stable, High-Conduction Solid Electrolytes of Arsenic-Substituted Li_4SnS_4. *Energy Environ. Sci.* **7**, 1053–1058 (2014).

40. Zhang, Z. *et al.* $Li_{4-x}Sb_xSn_{1-x}S_4$ Solid Solutions for Air-Stable Solid Electrolytes. *J. Energy Chem.* **41**, 171–176 (2020).

41. Zhou, Y. *et al.* Observation of Interfacial Degradation of Li_6PS_5Cl against Lithium Metal and $LiCoO_2$ via In Situ Electrochemical Raman Microscopy. *Batter. Supercaps* **3**, 647–652 (2020).

42. Jones, L. A. H. *et al.* Sn 5s^2 lone pairs and the electronic structure of tin sulphides: A

photoreflectance, high-energy photoemission, and theoretical investigation. *Phys. Rev. Mater.* **4**, 074602 (2020).

43. Steudel, R., Jensen, D. & Plinke, B. Low Temperature Raman Spectra of Dichlorosulfane (SCl_2), Tetrachlorosulfurane (SCl_4), Dichlorodisulfane (S_2Cl_2) and Dichlorodiselane (Se_2Cl_2). *Z. Naturforsch. B* **42**, 163–168 (1987).
44. Waugh, J. S. & Fedin, E. I. Determination of Hindered-Rotation Barriers in Solids. *Sov. Physics-Solid State* **4**, 1633–1636 (1963).
45. Wang, Y. *et al.* Design Principles for Solid-State Lithium Superionic Conductors. *Nat. Mater.* **14**, 1026–1031 (2015).
46. Pan, L. *et al.* Revisiting the Ionic Diffusion Mechanism in Li_3PS_4 via the Joint Usage of Geometrical Analysis and Bond Valence Method. *J. Materiomics* **5**, 688–695 (2019).
47. Al-Qawasmeh, A., Howard, J. & Holzwarth, N. A. W. Li_4SnS_4 and Li_4SnSe_4: Simulations of Their Structure and Electrolyte Properties. *J. Electrochem. Soc.* **164**, A6386 (2017).
48. Minafra, N., Culver, S. P., Li, C., Senyshyn, A. & Zeier, W. G. Influence of the Lithium Substructure on the Diffusion Pathways and Transport Properties of the Thio-LISICON $Li_4Ge_{1-x}Sn_xS_4$. *Chem. Mater.* **31**, 3794–3802 (2019).
49. Leube, B. T. *et al.* Lithium Transport in $Li_{4.4}M_{0.4}M'_{0.6}S_4$ (M = Al^{3+}, Ga^{3+}, and M' = Ge^{4+}, Sn^{4+}): Combined Crystallographic, Conductivity, Solid State NMR, and Computational Studies. *Chem. Mater.* **30**, 7183–7200 (2018).
50. Lutz, H. D., Schmidt, W. & Haeuseler, H. Chloride Spinels: A New Group of Solid Lithium Electrolytes. *J. Phys. Chem. Solids* **42**, 287–289 (1981).
51. Kanno, R., Takeda, Y. & Yamamoto, O. Ionic Conductivity of Solid Lithium Ion Conductors with the Spinel Structure: Li_2MCl_4 (M = Mg, Mn, Fe, Cd). *Mater. Res. Bull.* **16**, 999–1005 (1981).

52. Shannon, R. D. Revised Effective Ionic Radii and Systematic Studies of Interatomic Distances in Halides and Chalcogenides. *Acta Cryst. A* **32**, 751–767 (1976).
